# Multi-omics reveals specific host metabolism-microbiome associations in intracerebral hemorrhage

**DOI:** 10.3389/fcimb.2022.999627

**Published:** 2022-12-22

**Authors:** Lei Chen, Sai Wang, Yupeng Zhang, Ye Li, Xiangbin Zhang, Junyi Ma, Xuelun Zou, TianXing Yao, Si Li, Junyou Chen, Huifang Zhou, Lianxu Wu, Yanhong Zhou, Le Zhang

**Affiliations:** ^1^ Department of Neurology, Multi-Modal Monitoring Technology for Severe Cerebrovascular Disease of Human Engineering Research Center, National Clinical Research Center for Geriatric Disorders, Xiangya Hospital, Central South University, Changsha, Hunan, China; ^2^ Cancer Research Institute, Basic School of Medicine, Central South University, Changsha, Hunan, China

**Keywords:** intracerebral hemorrhage, gut microbiome, metagenomic, metabolomics, metabolites

## Abstract

Intracerebral hemorrhage (ICH) is the most devastating subtype of stroke, but effective prevention and treatment strategies are lacking. Recently, gut microbiome and its metabolitesis are considered to be an influencing factor of stroke. However, little is known about the effects of the gut microbiome on ICH and host metabolic activity. Therefore, we used 16S sequencing, macrogenomics sequencing and untargeted metabolomics to explore the differences in gut microbial-metabolome interactions between patients with intracerebral hemorrhage and healthy control populations. We found a significant decrease in the phylum of *Firmicutes* and a significant increase of Bacteroidetes in ICH patients. At the genus level, *Streptococcus, Bifidobacterium, Akkermansia*, and *Lactobacillus* were more abundant in ICH patients. Macrogenomic analysis revealed active glycosaminoglycan degradation, heme synthesis, galactose degradation, lipopolysaccharide core region synthesis, and beta-Lactam resistance in ICH patients. Serum untargeted metabolomic analysis combined with ROC curves showed that octanoylcarnitine, decanoylcarnitine, dodecanoylcarnitine, glyceric acid, pyruvic acid, aspartic acid, methylcysteine, pyroglutamic acid, 9E-tetradecenoic acid, N-Acetylneuraminic acid, and aconitic acid were the best markers for the diagnosis of ICH. Correlation analysis showed that microbiome enriched in the gut of ICH patients were significantly correlated with serum metabolites, revealing a close correlation between the gut microbiome of ICH patients and the host metabolome, and significant differences from the healthy population. microbiota-host co-metabolites including pyruvic acid and 9E-tetradecenoic acid is associated with the the National Institutes of Health Stroke Scale (NIHSS) scores. In conclusion, microbiome-related metabolites in ICH patients was associated with the severity of ICH, the microbiota-host co-metabolites may be a potential may be potential therapeutic targets.

## 1 Introduction

Intracerebral hemorrhage (ICH) is the most important stroke subtype with the highest mortality and disability rate, accounting for approximately 1/5 of stroke cases. ICH has serious negative impact on human health and imposes a tremendous economic burden on families and the society (Gross et al., 2019). With aging of the population, the incidence of cerebral hemorrhage is increasing annually. Therefore, it is important to find key targets for interventions of ICH.

The gut microbiome is involved in triggering and subsequent development of autoimmune diseases (Shahi et al., 2017; Franzosa et al., 2019), metabolic disorders (Ma et al., 2020; Wu et al., 2021), and neurodegenerative diseases (Qian et al., 2021; Wang et al., 2021). Studies revealed that the gut microbiome is associated with ischemic stroke. Chang et al. observed that gut microbial diversity and abundance of specific bacteria, such as *Aerococcaceae* and *Flavobacterium*, decreased in ischemic stroke (Chang et al., 2021). Another prospective study reported an increase in trimethylamine N-oxide (TMAO)-producing bacteria and reduction of butyrate-producing bacteria in ischemic stroke (Haak et al., 2021). Further experiments confirmed that short-chain fatty acid (SCFA)-producing bacteria alleviated post-stroke neurological deficits and inflammation, and increased SCFA concentrations, suggesting that microbiome-derived metabolites play an important role in the development of stroke. Benakis et al. demonstrated that the gut microbiome regulates the immune system and affects the outcome of acute brain injury in stroke mice (Benakis et al., 2016). In addition, TMAO (a microbial-derived metabolite) was significantly higher in the cohort of patients with ICH than in HCs and was an independent predictor of poor prognosis (Yu et al., 2021; Zhai et al., 2021). Recent evidence has shown that gut microbiome dysbiosis after ICH promotes neuroinflammation by affecting T cell homeostasis, and transplantation of healthy flora alleviates neuroinflammation and improves neurological outcomes (Yu et al., 2021). In conclusion, although the gut microbiome is a potential influencing factor of ICH, systematic studies regarding the composition of the gut microbiome and the contribution of metabolites in ICH are limited.

In this study, to decode the ICH-related functional and metabolic features of gut microbiome dysbiosis, we performed integrated microbiome and metabonomics analysis in the cohort of patients with ICH and identified ICH-related microbial species, functional pathways, and metabolites.

## 2 Materials and methods

### 2.1 Research participants and sample collection

We included patients diagnosed with acute cerebral hemorrhage at Xiangya Hospital of Central South University between September 2020 and January 2021 (n=31) and age- and sex-matched healthy controls (HCs, n=31).Patients were confirmed cerebral hemorrhage through head CT and/or MRI (according to the diagnostic criteria of the 4th National Conference on Cerebrovascular Diseases). Exclusion criteria including (1) use of antibiotics, probiotics or probiotics within 1 month before the collection of samples; (2) more than 3 weeks after the onset of the disease; (3) multiple organ failure or death within 3 weeks after the disease; (4) traumatic cerebral hemorrhage, cerebral amyloid angiopathy, secondary cerebral hemorrhage (aneurysm, vascular malformation, moyamoya disease, coagulation disease, tumor stroke, vasculitis, cerebral venous thrombosis, hemorrhagic cerebral infarction, etc.); (5) complicated with neurodegenerative diseases, mental diseases, hepatorenal failure, autoimmune diseases, malignant tumors, thyroid diseases and pregnancy.

We recruited healthy controls (HCs) from Physical Examination Department of Xiangya Hospital of Central South University. Inclusion criteria including (1) age 18-80 years old; (2) healthy volunteers in Hunan Province; (3) matching sex and age of patients with intracerebral hemorrhage; (4) signing informed consent. Exclusion criteria: (1) use of antibiotics or probiotics within one month before collection; (2) previous history of cerebrovascular diseases; (3) acute and chronic gastrointestinal diseases and previous gastrointestinal resection; (4) patients with neurodegenerative diseases, mental disorders, abnormal liver and kidney function, autoimmune diseases, malignant tumors, thyroid diseases and pregnancy.

Fresh fecal samples were collected and temporarily held in a liquid nitrogen or dry ice environment before being transported to a freezer and stored at -80°C. At the same time, the peripheral venous blood of the participants was collected, and the serum was immediately placed in a freezer at -80°C for cryopreservation. All sampling procedures were approved by the Medical Ethics Committee of Xiangya Hospital of Central South University. All participants signed written informed consent. (number: No.201912526).

### 2.2 Fecal DNA extraction for microbiome analysis

We used the E-Z 96^®^ Mag-Bind Soil DNA Kit (Omega, USA) to extract genomic DNA from feces, according to the manufacturer’s instructions. We used 1.2% agarose gel electrophoresis to verify DNA integrity and size, and spectrophotometry (Eppendorf, Germany) to determine DNA concentrations.

### 2.3 16S ribosomal RNA gene sequencing, taxonomic classification

DNA from 62 stool samples was sequenced and primers (pre-primer sequence 5, -ACTCCTACGGGAGGCAGCA-3, post-primer sequence 5, -GGACTACHVGGGTWTCTAAT-3) were used to amplify the V3 and V4 regions of the 16S rRNA gene. After sequencing library preparation, the MiSeq Reagent Kit V3 (Illumina, USA) was used to perform paired-end sequencing on a MiSeqPE300 sequencer (Illumina, USA). Raw sequences were quality-controlled, denoised, spliced, and dechimerized using QIIME2 to create a signature sequence (ASV) abundance table. Species annotation was performed on each signature sequence, based on the Greengenes database using QIIME2 software with default parameters.

### 2.4 Metagenomic sequencing and functional annotation

According to the manufacturer’s instructions, we used NovaSeq Reagent Kits to perform paired-end sequencing based on Illumina NovaSeq (Illumina Inc., San Diego, CA, USA). We used BLASTP to annotate representative sequences of non-redundant gene catalogs based on the NCBI-nr database. The KEGG and carbohydrate-active enzymes’ annotation was conducted using Diamond (http://www.diamondsearch.org/index.php, version 0.8.35) against the Kyoto Encyclopedia of Genes and Genomes database (http://www.genome.jp/keeg/, version 94.2) and hmmscan (http://hmmer.janelia.org/search/hmmscan) against the CAZy database (http://www.cazy.org/). The expected value of BLAST alignment parameter was set to 1e^-5^.

### 2.5 Bacterial diversity analysis

Using QIIME2 software, a sparse curve was drawn to evaluate the variation trend of alpha diversity with sequencing depth. Chao1, observed species, Shannon, Simpson, Faith’s phylogenic diversity (PD), Pielou’s evenness, Good’s coverage, and other alpha diversity indices were calculated; each alpha diversity index was evaluated. Bacterial diversity of each sample, principal coordinates analysis (PCoA) based on Jaccard’s difference index, was used to evaluate beta diversity between samples, and permutation multivariate analysis of variance (PERMANOVA) was used to test for significant differences between groups.

### 2.6 Statistical analysis

Serum metabolomics analysis based on PLC-MS/MS : Untargeted metabolomic detection of 62 human fecal and serum samples was performed using ultra-high-performance liquid chromatography-tandem mass spectrometry (PLC-MS/MS) (Waters Corp, Milford, MA, USA). We used the MassLynx software (v4.1, Waters, Milford, MA, USA) to process resulting raw data files for peak integration, calibration, and quantification of each metabolite. iMAP software (version 1.0, Metabo-Profile, Shanghai, China) was used for principal component, orthogonal partial least squares discriminant, one-dimensional statistical, and pathway analyses. Dimensionality reduction of metabolomic data was conducted using PCA to describe and visualize differences between samples based on distance matrices. Further Orthogonal Projections to Latent Structures Discriminant Analysis (OPLS-DA) modeling was performed to visualize the differences in metabolic profiles between the two groups, and metabolites with VIP >1 were significantly different between the groups. The *t*-test or Mann–Whitney U-test was used to test for differences in the abundance of metabolites between groups based on the normality and homogeneity of variance of the abundance data.Differential metabolites were screened according to single-dimensional statistical analysis P<0.05 and VIP>1 (OPLS-DA). The differential metabolites were retrieved using the KEGG library combined with pathway enrichment and pathway topology analyses, to calculate the P-value of differential metabolites aggregated in each pathway and the degree of impact on the metabolic pathway.

Statistical analysis of taxonomic features was performed using the R package to show species composition at different taxonomic levels. The Kruskal–Wallis rank-sum test was used to determine whether the difference in the alpha diversity index between the two groups was significant. Species/function difference analysis was performed using the linear discriminant analysis effect size (LEfSe) method with α set to 0.05, and the linear discriminant analysis(LDA) score set to >2. Based on the KEGG Orthology (KO) database (https://www.kegg.jp/kegg/ko.Html), we mapped different KOs to the corresponding modules, and modules with at least 2 KO mappings were provided. Spearman correlation calculations were performed using the R package (4.0.2) and visualized using the R package or Networkx package.

## 3 Results

### 3.1 Gut microbiome characterization in ICH

**Figure 1:**
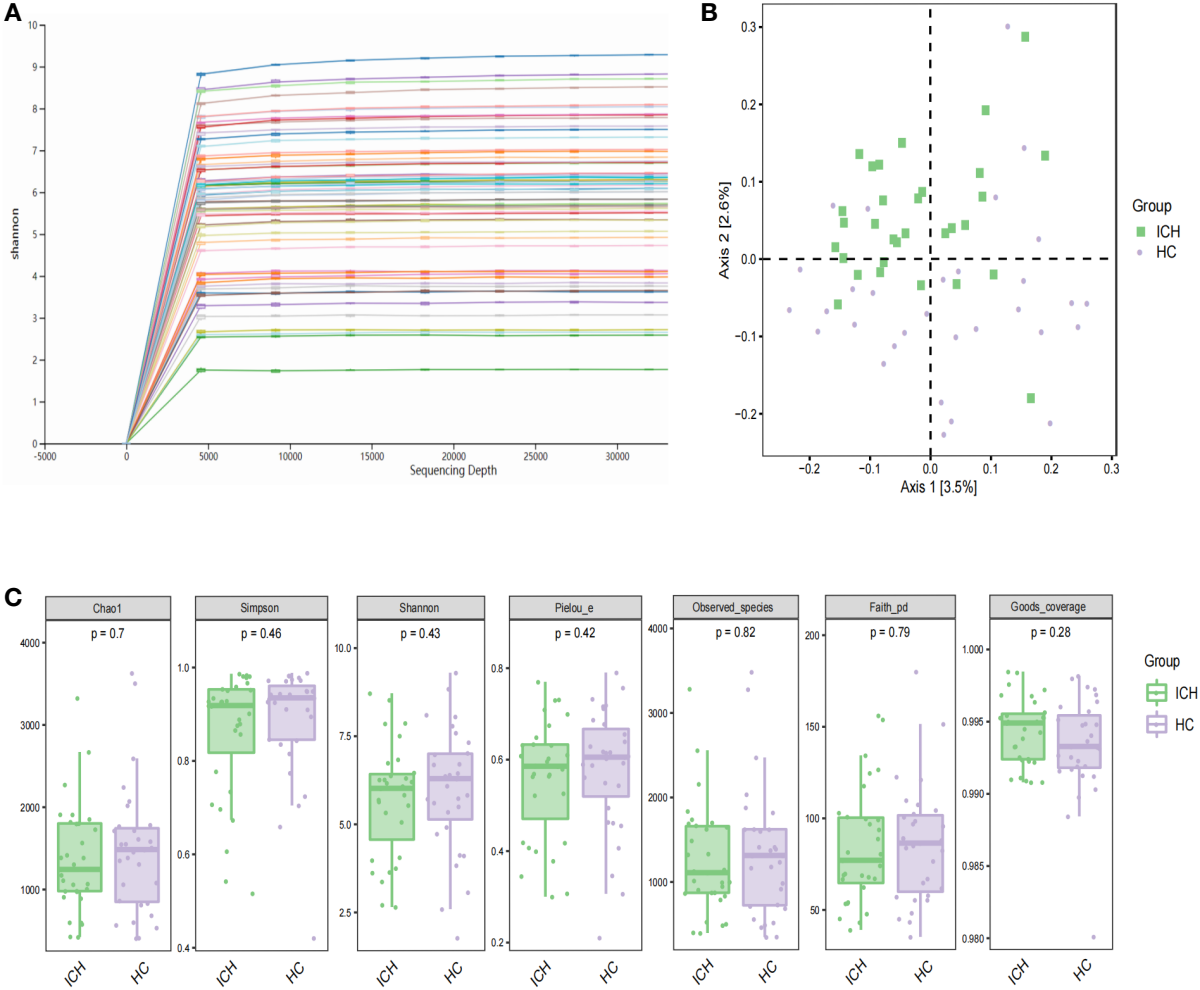
Characteristics and diversity of fecal microbiota. **(A)** Rarefaction curves; **(B)** PCoA based on Jaccard difference index shows the diversity of (β) between the two groups. Green squares and purple circles represent ICH and HC groups, respectively (PERMANOVA, P= 0.001); **(C)** box chart illustrating diversity index of Chao1, Simpson, Shannon and observed species in patients with ICH and HCs, respectively.

We performed 16S rDNA sequencing of fecal samples from 31 patients with newly diagnosed ICH and 31 sex - and age-matched HCs to explore the composition of the gut microbiome. The demographic and clinical information of the two groups were shown in **Table 1**. The majority of the bacterial read counts in the two groups were dominated by the phyla *Firmicutes*, *Bacteroidetes*, *Proteobacteria*, and *Actinobacteria*. In ICH, *Firmicutes*, *Bacteroidetes*, *Actinobacteria*, *Proteobacteria*, and *Verrucomicrobia* accounted for more than 99%, while the majority of gut microbiome in HCs belonged to *Firmicutes*, *Proteobacteria*, *Bacteroidetes*, *Actinobacteria*, and *Fusobacterium* (**Figure S1A**). At the genus level, the predominant gut microbiomes of the two groups were similar, but there were significant differences in abundance. *Bifidobacterium*, *Streptococcus*, *Bacteroides*, *Akkermansia*, *Lactobacillus*, and *Enterococcus* were more abundant in ICH, whereas *Faecalibacterium*, *Clostridium*, *Prevotella*, *Gemmiger*, and *Blautia* were more abundant in HCs (**Figure S1B**). **Figure 1A** shows rarefaction curves based on the Shannon index. With the increase in sequencing depth, the rarefaction curves of each sample gradually saturated, indicating that the sequencing depth of this experiment was sufficient to reflect the diversity of the current sample. We compared α diversity indices between the two groups. The results showed that there was no significant difference in the α-diversity index between the two groups, suggesting that there was no difference in the species richness, evenness, and diversity of intestinal microorganisms between the ICH and HC groups. We compared the alpha diversity indices (Chao1, observed species, Shannon, Simpson, Faith’s PD, Pielou’s evenness, Good’s coverage) between the two groups (**Figure 1B**) and observed no significant differences in the alpha diversity indices between patients with ICH and HCs (P>0.05), suggesting that species richness, evenness, and diversity of the gut microbiome were similar.

**Table 1:** Characteristics of the characteristics of the study participants.

	ICH patients (N=31)	HCs (N=31)	*P*
Mean age (years ± SD)	56.87 ± 10.246	52.65 ± 9.922	0.104
Male sex (n, %)	23 (74.19)	22 (70.97)	0.776
Smoking (n, %)	8 (25.81)	8 (25.81)	1.000
Alcohol-drinking (n, %)	11 (35.48)	8 (25.81)	0.409
NIHSS (mean, SD)	4.03, 4.215	—	

**Figure 2:**
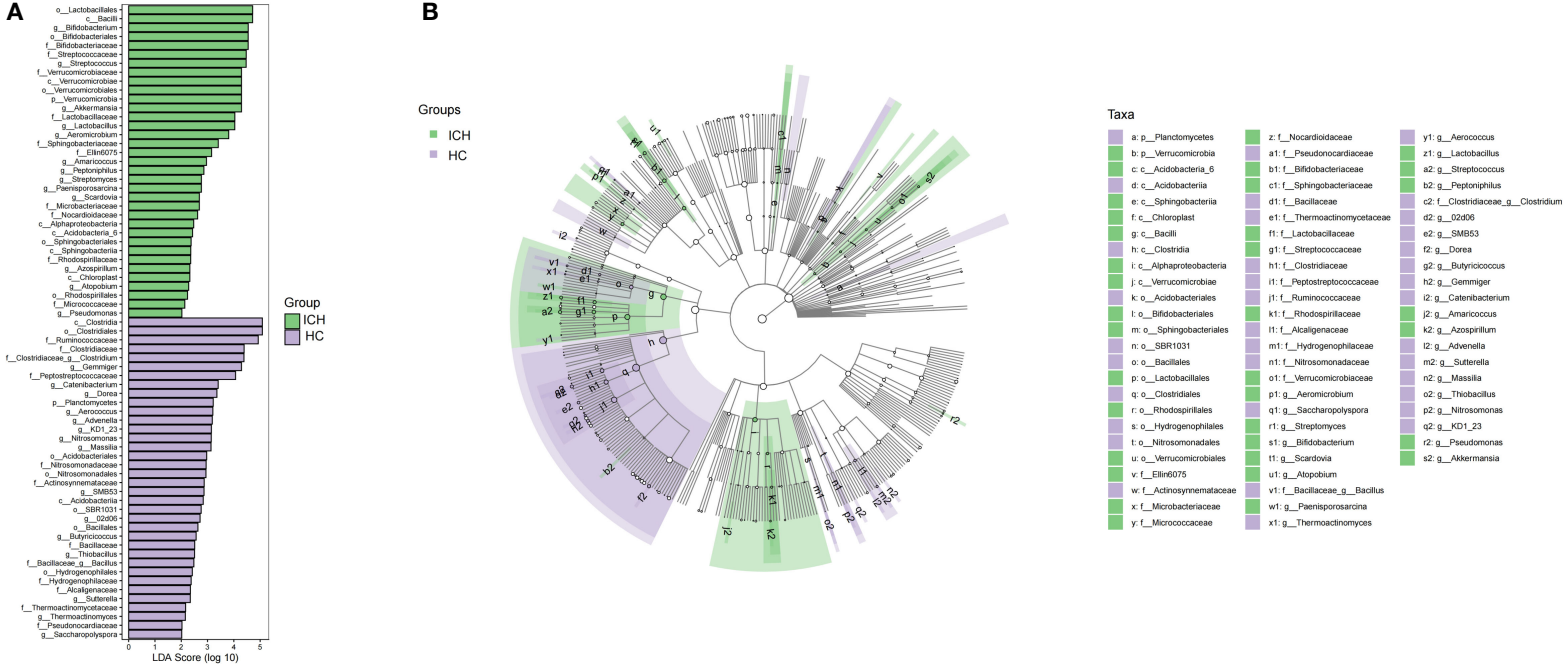
Varying and phylogenetic distribution of microbiota correlated with the ICH and HC groups. **(A)** Microbiota in significantly varying abundances (from phylum to genus level) between the ICH and HC groups; **(B)** Cladogram radiating from the inside to the outside representing the taxonomic level of microbiota from the phylum to the genus.

Next, we performed beta diversity analysis. PCoA based on the Jaccard dissimilarity index revealed that there were significant differences in the composition of the gut microbiome between the ICH and HC groups (**Figure 1C**). To further identify differential taxa, we used LEfSe and found 71 bacteria, of which 35 were enriched in ICH and 36 were enriched in HC. **Figure 2A** shows that *Lactobacillale*s, *Bacilli*, *Bifidobacterium*, *Bifidobacteriaceae*, *Bifidobacteriales*, S*treptococcaceae*, *Streptococcus*, *Verrucomicrobiaceae*, *Verrucomicrobiae*, *Verrucomicrobiales*, *Verrucomicrobia*, *Akkermansia*, *Lactobacillaceae*, and *Lactobacillus* were significantly enriched in ICH (LDA value (log10) >4). *Clostridia*, *Clostridiales*, *Ruminococcaceae*, *Clostridiaceae*, *Clostridium*, and *Peptostreptococcaceae* were the most abundant microbial groups in the HC group (LDA value (log10) >4). **Figure 2B** shows the evolutionary relationship of the differential microbiota between the groups. It could not be annotated using 16s rDNA sequencing. To further determine the different species, we analyzed the macrogenomes of 17 fecal samples. LEfSe analysis showed that *Akkermansia muciniphila*, *Escherichia coli*, and *Ruthenibacterium lactatiformans* were the most abundant in the ICH group at the species level (LDA value (log10) > 4), whereas *Faecalibacterium prausnitzii*, *Eubacterium rectale*, *Roseburia inulinivorans*, and *Anaerobutyricum hallii* were most abundant in the HC group (LDA value (log10) > 3.7) (**Figure S2**).

Based on the microbial abundance data, we constructed a microbial correlation network among the first 30 species in ICH, HCs, and all subjects, including ICH and HC, by calculating the Spearman correlation coefficient (**Figure S3**). The results showed more significant interactions between species in HCs than in ICH. We found that *Ruthenibacterium lactatiformans* had the highest node degree in ICH, which was negatively correlated with *Lactobacillus salivarius* and *Anaerostipes hadrus*, but positively correlated with Subdoligranulum_sp_4_3_54A2FAA, *Bifidobacterium pseudocatenulatum*, and *Clostridium cocleatum*. However, there was a difference in the HC group, which indicated a positive correlation between the core flora of *Faecalibacterium prausnitzii* and *Eubacterium rectale*. Most of the bacteria in HCs belonged to *Ruminococcaceae*, such as *Faecalibacterium prausnitzii*, *Subdoligranulum_sp*. _APC924/74, *Ruminococcus_bromii*, *Faecalibacterium_sp*, uncultured_*Ruminococcus* sp., and *Ruminococcus* sp., which were positively correlated with each other. In all participants (ICH and HC), *Faecalibacterium prausnitzii* and *Eubacterium rectale* were dominant in abundance and were positively correlated with each other, and *Escherichia coli* and *Akkermansia muciniphila* were significantly negatively correlated with *Eubacterium rectale*. We observed that there were significant differences in gut microbiome composition in patients with ICH compared to HCs group, but there was no significant difference in richness and diversity of the gut microbiota but there was no significant difference in richness and diversity of the gut microbiota. The core microbiota of ICH group were opportunistic pathogenic bacteria (*Ruthenibacterium lactatiformans*), whereas probiotics decreased. It is speculated that opportunistic pathogens overgrow and compete with the probiotics. Therefore, we believe that the pathological state of ICH group leads to gut dysbiosis. The poor interaction between gut microbiota is one of the characteristics of ICH group.

### 3.2 Alterations in function of fecal microbiome associated with ICH

**Figure 3:**
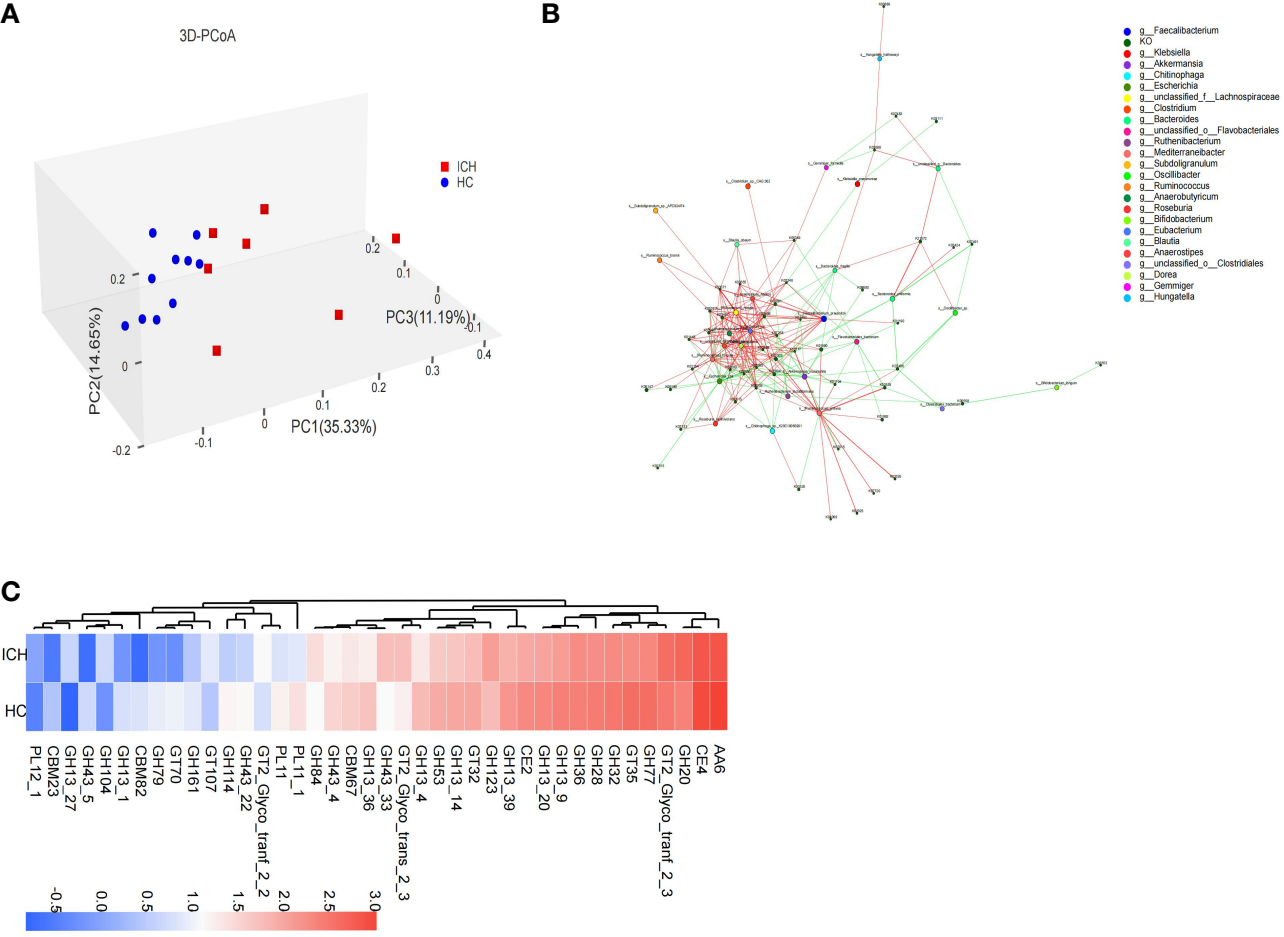
Functional characteristics of fecal microbiota in ICH. **(A)** PCoA (3D) based on Bray–Curtis difference index shows functional (KO) (β) diversity (PERMANOVA, P=0.002) between samples; **(B)** The correlation network diagram was constructed by the bacteria in the first 30 abundance and the KOs in the first 50 abundance, the red line represents positive correlation and the green line represents negative correlation **(C)** The difference in abundance of the CAZy family between the ICH and HC groups.

To explore the changes in gut microbial function in ICH group, we performed metagenomic sequencing on 17 stool samples and then annotated the obtained non-redundant gene set sequences using the KEGG gene database (GENES). KEGG integrates genomic, chemical, and systemic functional data. The abundance of KEGG Orthology (KO) was calculated based on the sum of the gene abundances corresponding to KO, and multiple analyses were performed. The Venn diagram (**Figure S4**) showed the shared and unique function KOs of ICH and HC group. In total, the HC group contained more KOs (HC: 6895 vs. ICH: 6275), and the two groups shared most of the KOs (87.6%). The top 50 most abundant KOs were selected, and sample and functional KO clustering were performed according to the abundance of KOs, thereby revealing the similarity or difference in KO abundance among different samples (**Figure S5**). To further compare the differences in functional composition between groups, PCoA based on the Bray–Curtis difference index showed that there were significant differences in the function (KO) of the gut microbiome between ICH and HC groups (**Figure 3A**).

By applying LEfSe, we identified 169 differential KOs, and most of the identified KOs were increased in abundance in HC group, indicating that the number of gut microbial KOs was reduced in ICH group. The identified differential KOs were mapped to 39 modules, of which 14 contained at least two KOs: cobalamin biosynthesis (M00122), F-type ATPase (M00157), dicarboxylate-hydroxybutyrate ester cycle (M00374), trehalose biosynthesis (M00565), glycogen biosynthesis (M00854), tricarboxylic acid cycle (M00009, M00011), shikimate pathway (M00022), ornithine biosynthesis (M00028), fatty acid biosynthesis synthesis (M00083), reduced citric acid cycle (M00173), incomplete reduction in the citric acid cycle (M00620), glycogen degradation (M00855), and molybdenum cofactor biosynthesis (M00880). Interestingly, all the modules were more abundant in the HC group. However, modules such as keratin sulfate degradation (M00079), heme biosynthesis (M00121), galactose degradation (M00632), ADP-L-glycero-D-manno-heptose biosynthesis (M00064), and β-lactam resistance (M00627) were more abundant in the ICH group. A correlation network map was constructed for the bacteria and KOs (**Figure 3B**). It was found that the ICH-enriched bacteria, such as *Akkermansia muciniphila* and *Escherichia coli*, were negatively correlated with most KOs, whereas the HC-enriched bacteria, such as *Faecalibacterium prausnitzii* and *Eubacterium rectale*, were positively correlated with most KOs, implying that gut microbial function decreased in ICH group.

To understand the carbohydrate-related metabolic function of gut microbiome, we analyzed the gene sequences after annotation, using the CAZy database. The results showed that there were significant differences in carbohydrate-active enzyme function between ICH and HC groups. The Venn diagram (**Figure S6**) showed that the number of CAZy enzyme genes in HCs was slightly higher than that in patients with ICH, and most of the enzymes (89.3%) were common between the two groups. PCoA showed that there was a significant difference in the classification of carbohydrate-active enzyme families between ICH and HC group (**Figure S7**). Eleven carbohydrate active enzyme family genes (6 GH,4 GT,1 PL), including GH123, GH20, and GT2_Glyco_tranf_2_3, were more abundant in ICH group, whereas 29 carbohydrate active enzyme family genes (18 GH,3 CBM,3 GT,2 CE,2 PL,1 AA), such as AA6, CE4, GT35, GH77, GH32, GH13_20, CE2, GH13_9, GH28, GH36, and GH13_39, were more abundant in the HC group (**Figure 3C**).

Compared to the HC group, the carbohydrate active enzyme family gene types of the gut microbiome in ICH group were significantly different, suggesting that gut microbiome in patients with ICH metabolize carbohydrates in different ways. Glycoside hydrolase (GH) was responsible for decomposing complex carbohydrates and glycoconjugates, and the number of GH annotations was the largest in the two groups, but the number of GH and carbohydrate-binding module (CBM) genes of the gut microbiome in ICH group decreased significantly. CBM plays an important role in cellulase degradation, suggesting a reduced cellulose degradation in ICH group.

### 3.3 ICH-specific serum metabolomes

**Figure 4:**
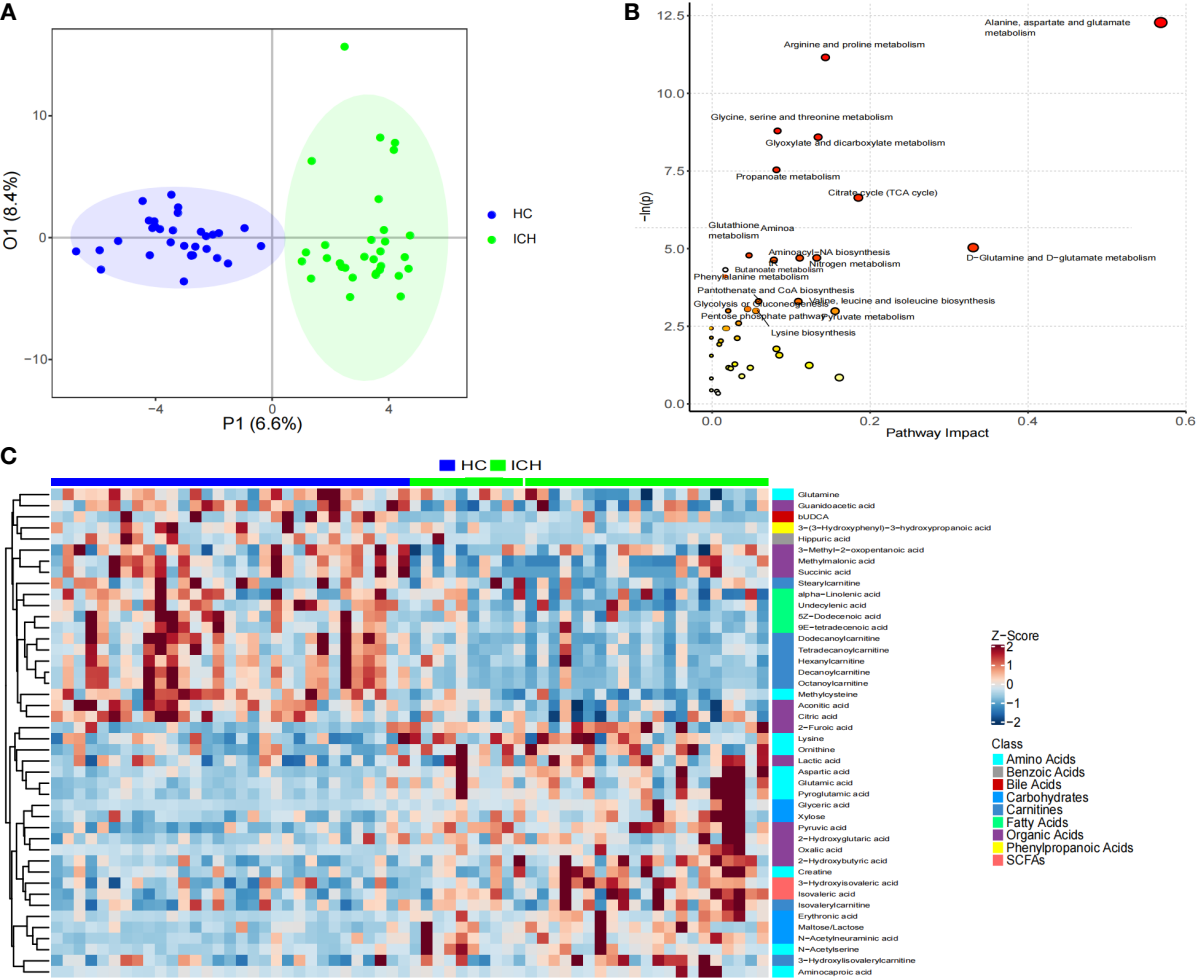
Characteristics of serum metabonomics. **(A)** OPLS-DA score chart; **(B)** Pathway analysis and statistical significance of differential metabolites in serum samples; **(C)** Heat maps showing relative abundances of differential metabolites screened using unidimensional and multidimensional statistics.

Based on the serum metabolomic data, we first used unsupervised PCA for analysis. We found a statistically significant difference between the two groups in the scores of PC1 (10.9%) and PC2 (9.6%) (0.024 and 0.042) (**Figure S8**), indicates the specific serum metabolomic of the ICH group. Further OPLS-DA analysis showed that there were significant differences in serum metabolomic characteristics between the ICH and HC groups (**Figure 4A**).

According to the distribution characteristics of the metabolite abundance data, the *t*-test or Mann–Whitney U test was used to determine significant differences between the groups. Sixty differential metabolites were obtained (**Figure S9**). Using the OPLS-DA model, 54 differential metabolites were screened according to VIP > 1 (**Figure S10**). Through the intersection of the results of the unidimensional and multidimensional statistical analyses, 44 differential metabolites were identified as potential biomarkers for predicting ICH (**Figure 4C**). These differential metabolites were organic acids, amino acids, carnitine, carbohydrates, fatty acids, short-chain fatty acids, benzoic acid, bile acid, and phenylpropionic acid. The main metabolites enriched in the ICH group were amino acids, organic acids, carbohydrates, and short-chain fatty acids, whereas those in the HC group were mainly organic acids, carnitine, fatty acids, benzoic acid, and phenylpropionic acid. For example, the abundance of pyruvic acid, maltose/lactose, erythronic acid, furoic acid, and aspartic acid increased significantly in the ICH group (log2FC > 1), whereas the abundance of succinic acid, hippuric acid, octanoylcarnitine, decanoylcarnitine, methylcysteine, undecenoic acid, and dodecylcarnitine increased significantly in the HC group (log2FC > 1).

By searching the KEGG database for pathway analysis, we found that serum differential metabolites were enriched in alanine, aspartic acid, and glutamic acid metabolism; arginine and proline metabolism; glycine, serine, and threonine metabolism; glyoxylic acid and dicarboxylic acid metabolism; propionic acid metabolism; tricarboxylic acid cycle; D-glutamine and D-glutamic acid metabolism; glutathione metabolism; nitrogen metabolism; aminoacyl-tRNA synthesis; and butyric acid metabolism; Phenylalanine metabolism; and other metabolic pathways (**Figure 4B**).

**Figure 5:**
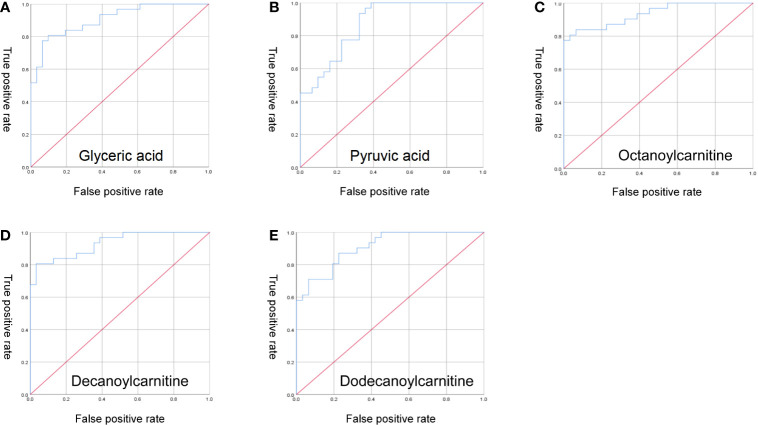
ROC curve analysis. **(A)** glyceric acid; **(B)** pyruvic acid; **(C)** octanoylcarnitine; **(D)** decanoylcarnitine; **(E)** dodecanoylcarnitine.

Performing ROC curve analysis of serum differential metabolites, we found that aspartic acid, glyceric acid, N-Acetylneuraminic acid, pyroglutamic acid, pyruvic acid, methylcysteine, aconitic acid 9E-tetradecenoic acid, octanoylcarnitine, decanoylcarnitine, dodecanoylcarnitine, tetradecanoylcarnitine are significant for the diagnosis of ICH. Especially, octanoylcarnitine, decanoylcarnitine, dodecanoylcarnitine, glyceric acid, pyruvic acid, the area under the curve was 0. 931, 0. 932, 0. 905, 0. 907, 0. 872 (**Figure 5**), and therefore would serve as the most important biomarkers.

### 3.4 Association analysis of gut differential microbiome and differential metabolites

**Figure 6:**
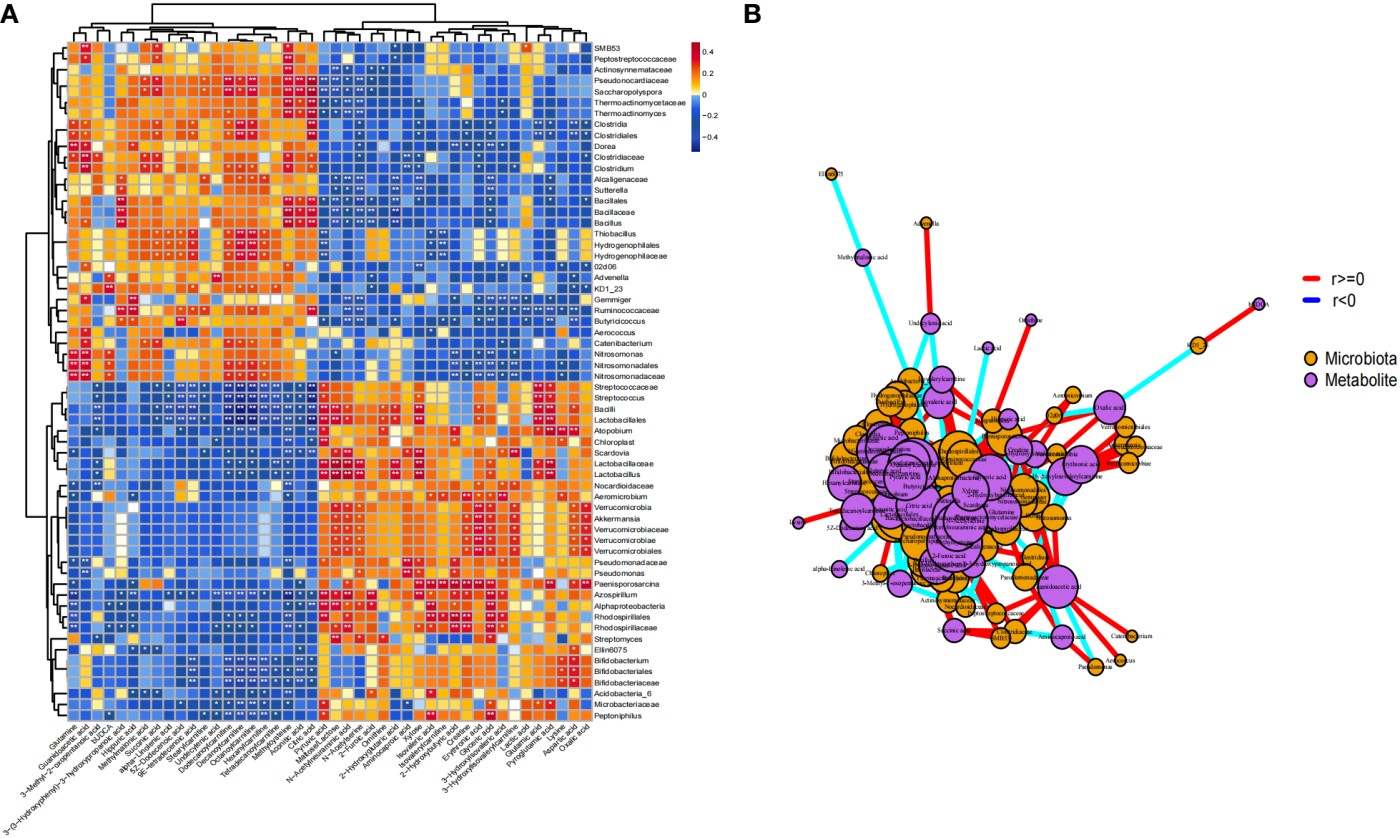
Comprehensive correlation network analysis of microbiota and metabolites in serum. **(A)** Heat map showing the Spearman correlation between different microbiota and different metabolites in stool samples (P < 0.05); **(B)** Network diagram. Purple nodes represent the metabolites of fecal samples, orange nodes represent microbiota. The size of nodes and the number of inter-node connections are linearly related. * p<0.05 ** p<0.01.

Based on the microbiome and metabolomic data, we calculated the Spearman correlation coefficient between the two groups to identify microbiome-related metabolites in ICH. We observed strong correlations between differential metabolites and microbes. Microbes that were more abundant in ICH were positively correlated with ICH-enriched metabolites but negatively with HC-enriched metabolites (**Figure 6A**), indicating that metabolic modes of ICH are highly associated with changes in the gut microbiome. We obtained signature microbe–metabolite association pairs and relationship chains from heatmap and network diagram (**Figure 6B**). For example, *Akkermansia* were significantly positively correlated with N-Acetylneuraminic acid and glyceric acid. *Lactobacillus* were significantly positively correlated with N-Acetylneuraminic acid and Pyruvic acid. *Streptococcus*, *Bifidobacterium* and *Lactobacillus* were significantly negatively correlated with octanoylcarnitine, decanoylcarnitine and dodecanoylcarnitine. Moreover, the microbe–metabolite association pairs and relationship chains are potential targets for future *in vivo*/*in vitro* experiments.

We used spearman’s correlation analysis to evaluate the potential relationship between the microbiome-related metabolites and the severity of ICH. Pyruvic acid and Maltose/Lactose were both positively correlated with NIHSS scores(r=0.412, p=0.021, and r=0.383, p=0.034, respectively). 9E-tetradecenoic, alpha-Linolenic acid and Stearylcarnitine were both negatively correlated with NIHSS scores(r=-0.433, p=0.015, r =-0.422, p=0.018, and r =-0.366, p=0.043,respectively). In conclusion, microbiome-related metabolites in ICH patients was associated with the severity of ICH.

## 4 Discussion

In this study, we identified the structural and functional characteristics of gut microbiome in patients with ICH, in which the dominant bacterial phyla were Firmicutes and Bacteroidetes, but the ratio of Firmicutes/Bacteroidetes was different compared to healthy population. Recently, The ratio of Firmicutes/Bacteroidetes is used as an indicator of gut microbial dysbiosis and has been reported to decrease in immune diseases such as systemic lupus erythematosus, rheumatoid arthritis, obesity, and type 1 diabetes (Kasselman et al., 2018). The metabolic endotoxemia hypothesis was then proposed, whereby pro-inflammatory molecules such as lipopolysaccharides enter the circulatory system through the intestinal barrier after prolonged exposure to Gram-negative bacteria (mostly belonging to the phylum Bacteroides), triggering endotoxemia and leading to a mild inflammatory state (Cani et al., 2007). Our macrogenomic results give further clues - the lipopolysaccharide core region biosynthesis is significantly increased. The relationship between gut microbiome and stroke inflammation continues to be demonstrated, with gut dysbiosis in stroke patients and secreted pro-inflammatory molecules activating the systemic inflammatory response, leading to increased neuroinflammation (Wang et al., 2022; Wang et al., 2022). Zheng Q et al. found that Klebsiella, Escherichia-Shigella, and Bacteroides were positively correlated with systemic inflammatory markers in stroke patients (Zheng et al., 2022). Mice received fecal bacteria transplantation from stroke patients or injection of Lipopolysaccharide exacerbated the neuroinflammatory response (Wang et al., 2022). Our findings further explain the above phenomenon that active synthesis of pro-inflammatory molecules by gut microbiome in ICH patients promotes a systemic inflammatory response, ultimately lead to increased neuroinflammation.

Disorders of gut microbiota-host co-metabolites are associated with diseases (Yang et al., 2019; Yan et al., 2022; Qian et al., 2022). Correlation analysis has shown a positive correlation between the pyruvic acid and the severity of ICH. Pyruvic acid is the metabolic end product of glycolysis, and accelerated glycolysis is involved in a variety of pathological states. Metabolomic analysis revealed a shift in metabolic patterns toward enhanced glycolysis in patients with subarachnoid hemorrhage (Gusdon et al., 2022). Administration of high doses of pyruvic acid to pMCAO mice increased cortical infarct volume and exacerbated neurological deficits (González-Falcón et al., 2003). Conversely, Inhibition of glycolysis reduced neuroinflammation. Accelerated glycolysis in LPS-induced pro-inflammatory glial cells, regulation of metabolic reprogramming inhibits neuroinflammation (Wang et al., 2022; Pan et al., 2022). Acylcarnitine is involved in fatty acid β-oxidation, and our study firstly discovered gut microbiome significantly associated with reduced serum Acylcarnitine in patients with ICH. Previous studies have reported altered fatty acid β-oxidation responses in stroke-prone spontaneously hypertensive rats (Tanaka et al., 2013). Acylcarnitine levels (short- and medium-chain acylcarnitine) were associated with immune responses (Waagsbø et al., 2016) and Inhibitors of fatty acid oxidation improved neuroinflammatory and demyelination in EAE animals, as well as improved symptoms of Huntington’s chorea (Bogie et al., 2020). Furthermore, our study showed that elevated N-acetylneuraminic acid levels in ICH were positively correlated with ICH-enriched species (*Akkermansia* et al). Blood N-acetylneuraminic acid and sialidase activity are elevated in patients with ischemic stroke (Nanetti et al., 2008) and N-acetylneuraminic acid levels increase the risk of death of cardiovascular disease and stroke (Lindberg et al., 1992; Li et al., 2021), but little known the mechanism. *Akkermansia muciniphila* is the most important species in *Verrucomicrobiota*, and were more significant enrichment in ICH. Two recent studies that included 10 patients each with parenchymal hemorrhage reported a significant increase in abundance of *Verrucomicrobiota* (Li et al., 2022; Xiong et al., 2022), which is consistent with our results. This bacteria uses mucin O-glycan, a major component of intestinal mucus, as the main carbon source and is sialidase-rich species, while excessive degradation of mucin by microbiome can exacerbate intestinal inflammation (Patel et al., 2022; Yao et al., 2022). The present study provides new perspective of gut microbiome, microbiome-host co-metabolites may be potential therapeutic targets.

In this study, Han patients with ICH and healthy individuals were selected as the research subjects in the Hunan area (Yang et al., 2004), a region known worldwide for a high incidence of ICH, to explore the specific relationship between the gut microbiome, metabolic activities and the host in ICH from a new perspective of the gut microbiome, thereby providing clues for the study of the pathogenesis of ICH. This study has several limitations. We applied non-targeted metabolomics analysis, whereas targeted metabolomics will be used in the future to verify the differential metabolites found. The study initially reported the association between the gut microbiome and metabolites in ICH, but no causal verification was conducted *in vivo*; fecal bacteria transplantation using an animal model of spontaneous ICH is required in the future. Further experiments are urgently needed to verify the influence of the characteristic gut microbes of ICH on the occurrence and development of ICH, and to explore the possible mechanism.

## Data availability statement

The original contributions presented in the study are included in the article/**Supplementary Materials**. Further inquiries can be directed to the corresponding authors.

## Ethics statement

The studies involving human participants were reviewed and approved by Medical Ethics Committee of Xiangya Hospital of Central South University. The patients/participants provided their written informed consent to participate in this study.

## Author contributions

The co-authors LC and SW contributed equally to this article in clinical recruitment, drafted the manuscript and statistical analysis. LZ and YHZ designed the study. YPZ, YL, XBZ, and JM collected the samples. XLZ, TY, and SL contributed to the reagents, materials, and analysis tools. JC, HZ, and LW provided technical assistance. All authors contributed to the article and approved the submitted version.

## References

[B1] Benakis C. Brea D. Caballero S. Faraco G. Moore J. Murphy M. . (2016). Commensal microbiota affects ischemic stroke outcome by regulating intestinal γδ T cells. Nat. Med. 22 (5), 516–523. doi: 10.1038/nm.4068 27019327 PMC4860105

[B2] Bogie J. F. J. Haidar M. Kooij G. Hendriks J. J. A. (2020). Fatty acid metabolism in the progression and resolution of CNS disorders. Adv. Drug Delivery Rev. 159, 198–213. doi: 10.1016/j.addr.2020.01.004 31987838

[B3] Cani P. D. Amar J. Iglesias M. A. Poggi M. Knauf C. Bastelica D. . (2007). Metabolic endotoxemia initiates obesity and insulin resistance. Diabetes 56 (7), 1761–1772. doi: 10.2337/db06-1491 17456850

[B4] Chang Y. Woo H. G. Jeong J. H. Kim G. H. Park K. D. Song T.-J. (2021). Microbiota dysbiosis and functional outcome in acute ischemic stroke patients. Sci. Rep. 11 (1), 10977. doi: 10.1038/s41598-021-90463-5 34040060 PMC8155119

[B5] Franzosa E. A. Sirota-Madi A. Avila-Pacheco J. Fornelos N. Haiser H. J. Reinker S. . (2019). Gut microbiome structure and metabolic activity in inflammatory bowel disease. Nat. Microbiol. 4 (2), 293–305. doi: 10.1038/s41564-018-0306-4 30531976 PMC6342642

[B6] González-Falcón A. Candelario-Jalil E. García-Cabrera M. León O. S. (2003). Effects of pyruvate administration on infarct volume and neurological deficits following permanent focal cerebral ischemia in rats. Brain Res. 990 (1-2), 1–7. doi: 10.1016/s0006-8993(03)03378-x 14568323

[B7] Gross B. A. Jankowitz B. T. Friedlander R. M. (2019). Cerebral intraparenchymal hemorrhage: A review. JAMA 321 (13), 1295–1303. doi: 10.1001/jama.2019.2413 30938800

[B8] Gusdon A. M. Fu C. Putluri V. Paz A. S. Chen H. Ren X. . (2022). Early systemic glycolytic shift after aneurysmal subarachnoid hemorrhage is associated with functional outcomes. Neurocrit. Care. 37(3), 724–734. doi: 10.1007/s12028-022-01546-8 35799091 PMC10473383

[B9] Haak B. W. Westendorp W. F. van Engelen T. S. R. Brands X. Brouwer M. C. Vermeij J.-D. . (2021). Disruptions of anaerobic gut bacteria are associated with stroke and post-stroke infection: a prospective case-control study. Transl. Stroke Res. 12 (4), 581–592. doi: 10.1007/s12975-020-00863-4 33052545 PMC8213601

[B10] Kasselman L. J. Vernice N. A. DeLeon J. Reiss A. B. (2018). The gut microbiome and elevated cardiovascular risk in obesity and autoimmunity. Atherosclerosis 271, 203–213. doi: 10.1016/j.atherosclerosis.2018.02.036 29524863

[B11] Lindberg G. Råstam L. Gullberg B. Eklund G. A. (1992). Serum sialic acid concentration predicts both coronary heart disease and stroke mortality: multivariate analysis including 54,385 men and women during 20.5 years follow-up. Int. J. Epidemiol. 21 (2), 253–257. doi: 10.1093/ije/21.2.253 1428477

[B12] Li W. Wu L.-X. Huang B.-S. Yang L.-J. Huang J.-Q. Li Z.-S. . (2022). A pilot study: Gut microbiota, metabolism and inflammation in hypertensive intracerebral haemorrhage. J. Appl. Microbiol. 133 (2), 972–986. doi: 10.1111/jam.15622 35560738

[B13] Li C. Zhao M. Xiao L. Wei H. Wen Z. Hu D. . (2021). Prognostic value of elevated levels of plasma n-acetylneuraminic acid in patients with heart failure. Circ. Heart Fail 14 (11), e008459. doi: 10.1161/CIRCHEARTFAILURE.121.008459 34711067

[B14] Ma L. Ni Y. Wang Z. Tu W. Ni L. Zhuge F. . (2020). Spermidine improves gut barrier integrity and gut microbiota function in diet-induced obese mice. Gut Microbes 12 (1), 1–19. doi: 10.1080/19490976.2020.1832857 PMC7668533 33151120

[B15] Nanetti L. Vignini A. Raffaelli F. Taffi R. Silvestrini M. Provinciali L. . (2008). Sialic acid and sialidase activity in acute stroke. Dis. Markers 25 (3), 167–173. doi: 10.1155/2008/613272 19096129 PMC3827798

[B16] Pan R.-Y. He L. Zhang J. Liu X. Liao Y. Gao J. . (2022). Positive feedback regulation of microglial glucose metabolism by histone H4 lysine 12 lactylation in alzheimer's disease. Cell Metab. 34 (4), 634–648.e6. doi: 10.1016/j.cmet.2022.02.013 35303422

[B17] Patel V. C. Lee S. McPhail M. J. W. Da Silva K. Guilly S. Zamalloa A. . (2022). Rifaximin-α reduces gut-derived inflammation and mucin degradation in cirrhosis and encephalopathy: RIFSYS randomised controlled trial. J. Hepatol. 76 (2), 332–342. doi: 10.1016/j.jhep.2021.09.010 34571050

[B18] Qian X.-H. Song X.-X. Liu X.-L. S-d C. Tang H.-D. (2021). Inflammatory pathways in alzheimer's disease mediated by gut microbiota. Ageing Res. Rev. 68, 101317. doi: 10.1016/j.arr.2021.101317 33711509

[B19] Qian X. Zhang H.-Y. Li Q.-L. Ma G.-J. Chen Z. Ji X.-M. . (2022). Integrated microbiome, metabolome, and proteome analysis identifies a novel interplay among commensal bacteria, metabolites and candidate targets in non-small cell lung cancer. Clin. Transl. Med. 12 (6), e947. doi: 10.1002/ctm2.947 35735103 PMC9218934

[B20] Shahi S. K. Freedman S. N. Mangalam A. K. (2017). Gut microbiome in multiple sclerosis: The players involved and the roles they play. Gut Microbes 8 (6), 607–615. doi: 10.1080/19490976.2017.1349041 28696139 PMC5730390

[B21] Tanaka S. Kojiguchi C. Yamazaki T. Mitsumoto A. Kobayashi D. Kudo N. . (2013). Altered fatty acid profile in the liver and serum of stroke-prone spontaneously hypertensive rats: reduced proportion of cis-vaccenic acid. J. Oleo Sci. 62 (11), 933–948. doi: 10.5650/jos.62.933 24200942

[B22] Waagsbø B. Svardal A. Ueland T. Landrø L. Øktedalen O. Berge R. K. . (2016). Low levels of short- and medium-chain acylcarnitines in HIV-infected patients. Eur. J. Clin. Invest. 46 (5), 408–417. doi: 10.1111/eci.12609 26913383

[B23] Wang Q. Luo Y. Ray Chaudhuri K. Reynolds R. Tan E.-K. Pettersson S. (2021). The role of gut dysbiosis in parkinson's disease: mechanistic insights and therapeutic options. Brain 144 (9), 2571–2593. doi: 10.1093/brain/awab156 33856024

[B24] Wang X. Sun L. Guan S. Yan H. Huang X. Liang M. . (2022). Cyclin-dependent kinase 5 inhibitor attenuates lipopolysaccharide-induced neuroinflammation through metabolic reprogramming. Eur. J. Pharmacol. 929, 175118. doi: 10.1016/j.ejphar.2022.175118 35787890

[B25] Wang H. Zhang M. Li J. Liang J. Yang M. Xia G. . (2022). Gut microbiota is causally associated with poststroke cognitive impairment through lipopolysaccharide and butyrate. J. Neuroinflamm. 19 (1), 76. doi: 10.1186/s12974-022-02435-9 PMC8981610 35379265

[B26] Wang J. Zhong Y. Zhu H. Mahgoub O. K. Jian Z. Gu L. . (2022). Different gender-derived gut microbiota influence stroke outcomes by mitigating inflammation. J. Neuroinflamm. 19 (1), 245. doi: 10.1186/s12974-022-02606-8 PMC9531521 36195899

[B27] Wu J. Wang K. Wang X. Pang Y. Jiang C. (2021). The role of the gut microbiome and its metabolites in metabolic diseases. Protein Cell 12 (5), 360–373. doi: 10.1007/s13238-020-00814-7 33346905 PMC8106557

[B28] Xiong Z. Peng K. Song S. Zhu Y. Gu J. Huang C. . (2022). Cerebral intraparenchymal hemorrhage changes patients' gut bacteria composition and function. Front. Cell Infect. Microbiol. 12, 829491. doi: 10.3389/fcimb.2022.829491 35372117 PMC8966894

[B29] Yan J. Chen Q. Tian L. Li K. Lai W. Bian L. . (2022). Intestinal toxicity of micro- and nano-particles of foodborne titanium dioxide in juvenile mice: Disorders of gut microbiota-host co-metabolites and intestinal barrier damage. Sci. Total Environ. 821, 153279. doi: 10.1016/j.scitotenv.2022.153279 35074372

[B30] Yang Y. Misra B. B. Liang L. Bi D. Weng W. Wu W. . (2019). Integrated microbiome and metabolome analysis reveals a novel interplay between commensal bacteria and metabolites in colorectal cancer. Theranostics 9 (14), 4101–4114. doi: 10.7150/thno.35186 31281534 PMC6592169

[B31] Yang Q.-D. Niu Q. Zhou Y.-H. Liu Y.-H. Xu H.-W. Gu W.-P. . (2004). Incidence of cerebral hemorrhage in the changsha community. Cerebrovascular Dis. 17(4), 303–313. doi: 10.1159/000077341 15026613

[B32] Yao Y. Kim G. Shafer S. Chen Z. Kubo S. Ji Y. . (2022). Mucus sialylation determines intestinal host-commensal homeostasis. Cell 185 (7), 1172–1188.e28. doi: 10.1016/j.cell.2022.02.013 35303419 PMC9088855

[B33] Yu X. Zhou G. Shao B. Zhou H. Xu C. Yan F. . (2021). Gut microbiota dysbiosis induced by intracerebral hemorrhage aggravates neuroinflammation in mice. Front. Microbiol. 12, 647304. doi: 10.3389/fmicb.2021.647304 34025607 PMC8137318

[B34] Zhai Q. Sun T. Sun C. Yan L. Wang X. Wang Y. . (2021). High plasma levels of trimethylamine n-oxide are associated with poor outcome in intracerebral hemorrhage patients. Neurol. Sci. 42 (3), 1009–1016. doi: 10.1007/s10072-020-04618-9 32705490

[B35] Zheng Q. Chen Y. Zhai Y. Meng L. Liu H. Tian H. . (2022). Gut dysbiosis is associated with the severity of cryptogenic stroke and enhanced systemic inflammatory response. Front. In Immunol. 13, 836820. doi: 10.3389/fimmu.2022.836820 35603160 PMC9120915

