# Supplementary material for: Multi-omics reveals specific host metabolism-microbiome associations in intracerebral hemorrhage
Source: Front Cell Infect Microbiol. 2022 Dec 22;12:999627. doi: 10.3389/fcimb.2022.999627 (PMC9813413; doi:10.3389/fcimb.2022.999627)
Supplement: Supplementary file 1 [file DataSheet_1.pdf]

Figure S1

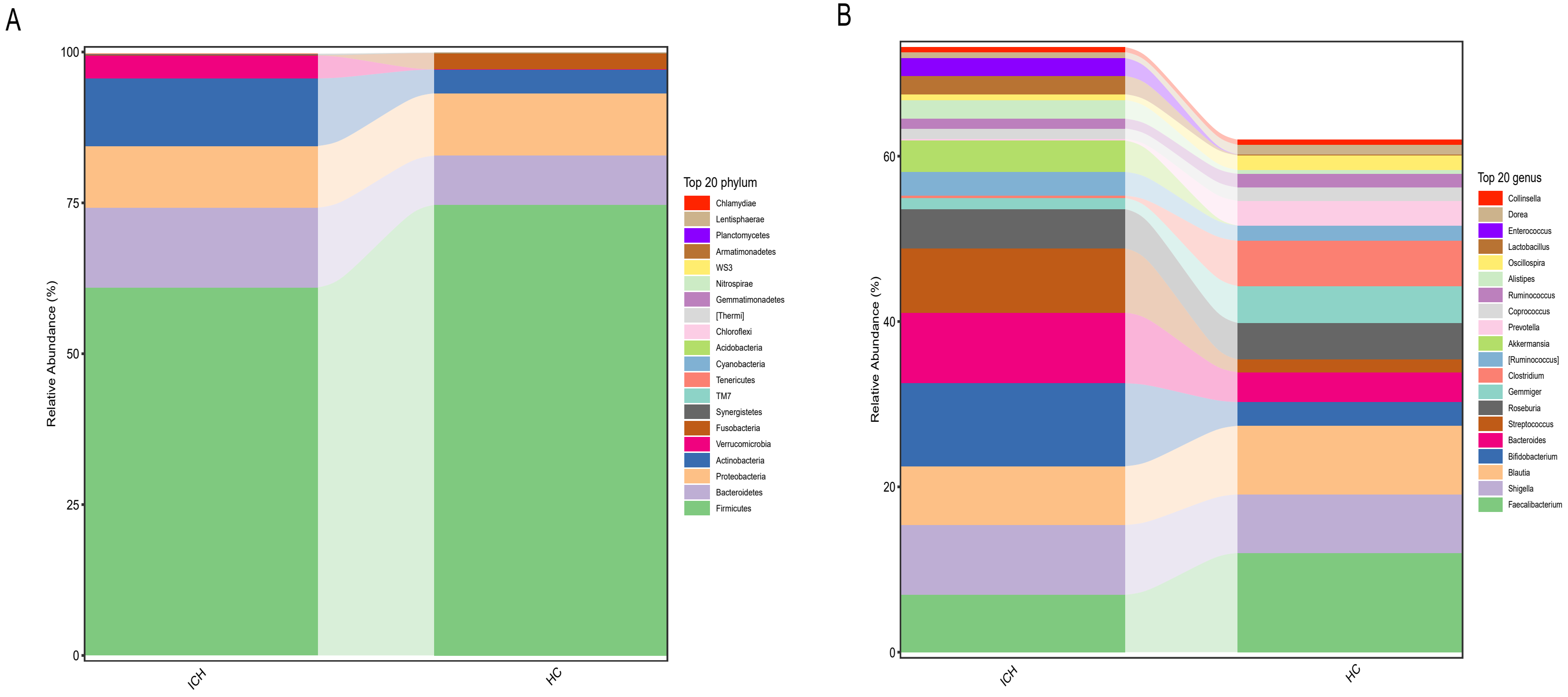
A
B
Relative Abundance (%)
100
75
50
25
0
ICH
HC
Top 20 phylum
Chlamydiae
Lentisphaerae
Planctomycetes
Armatimonadetes
WS3
Nitrospirae
Gemmatimonadetes
[Thermi]
Chloroflexi
Acidobacteria
Cyanobacteria
Tenericutes
TM7
Synergistetes
Fusobacteria
Verrucomicrobia
Actinobacteria
Proteobacteria
Bacteroidetes
Firmicutes
Relative Abundance (%)
60
40
20
0
ICH
HC
Top 20 genus
Collinsella
Dorea
Enterococcus
Lactobacillus
Oscillospira
Alistipes
Ruminococcus
Coprococcus
Prevotella
Akkermansia
[Ruminococcus]
Clostridium
Gemmiger
Roseburia
Streptococcus
Bacteroides
Bifidobacterium
Blautia
Shigella
Faecalibacterium

Figure S2

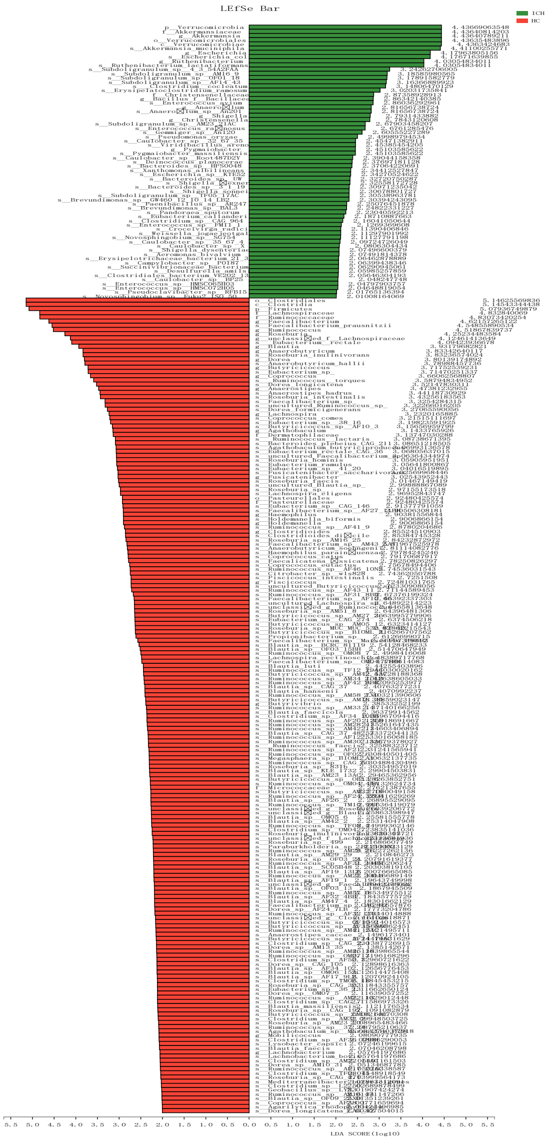

Figure S3

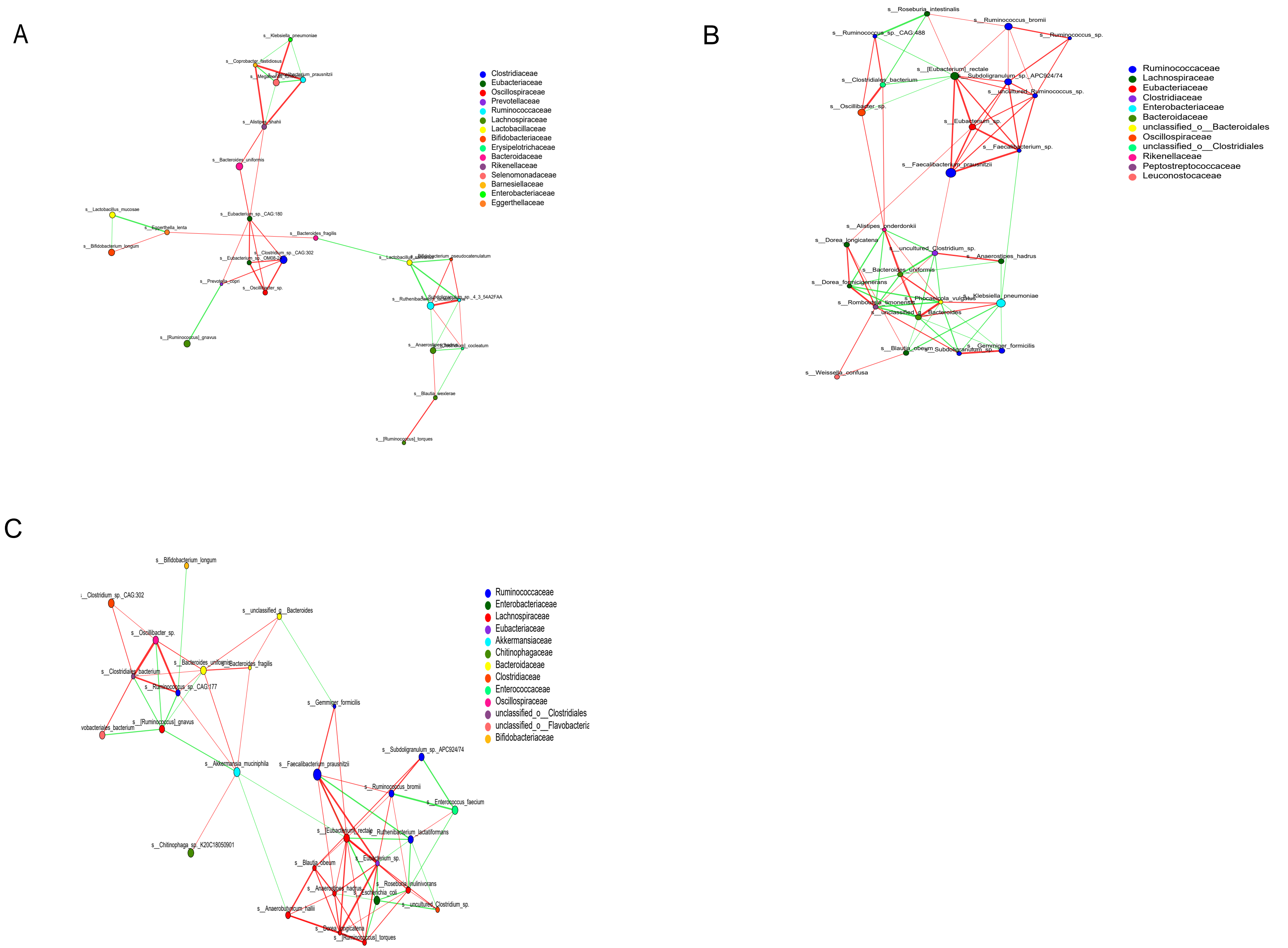
A
Clostridiaceae
Eubacteriaceae
Oscillospiraceae
Prevotellaceae
Ruminococcaceae
Lachnospiraceae
Lactobacillaceae
Bifidobacteriaceae
Erysipelotrichaceae
Bacteroidaceae
Rikenellaceae
Selenomonadaceae
Barnesiellaceae
Enterobacteriaceae
Eggerthellaceae
B
Ruminococcaceae
Lachnospiraceae
Eubacteriaceae
Clostridiaceae
Enterobacteriaceae
Bacteroidaceae
unclassified_o__Bacteroidales
Oscillospiraceae
unclassified_o__Clostridiales
Rikenellaceae
Peptostreptococcaceae
Leuconostocaceae
C
Ruminococcaceae
Enterobacteriaceae
Lachnospiraceae
Eubacteriaceae
Akkermansiaceae
Chitinophagaceae
Bacteroidaceae
Clostridiaceae
Enterococcaceae
Oscillospiraceae
unclassified_o__Clostridiales
Bifidobacteriaceae

Figure S4

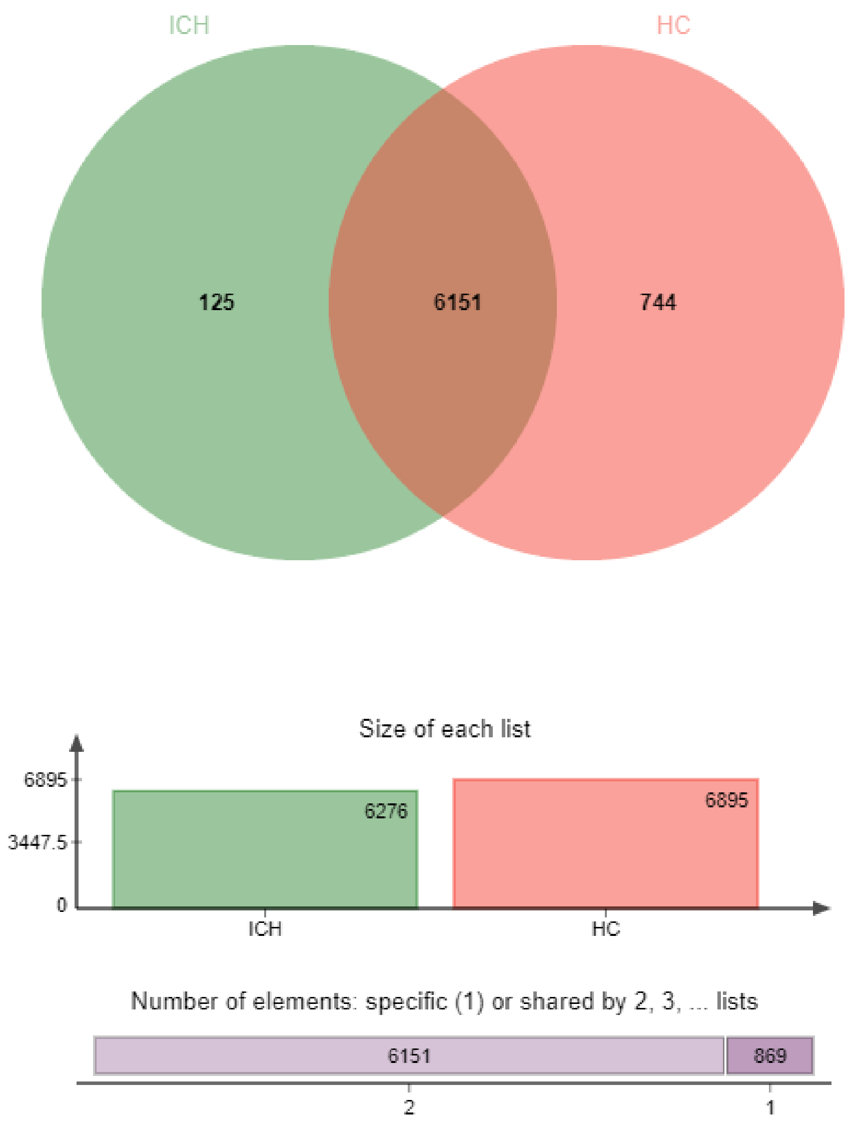
ICH
HC
125
6151
744
Size of each list
6895
3447.5
0
6276
6895
ICH
HC
Number of elements: specific (1) or shared by 2, 3, ... lists
6151
869
2
1

Figure S5

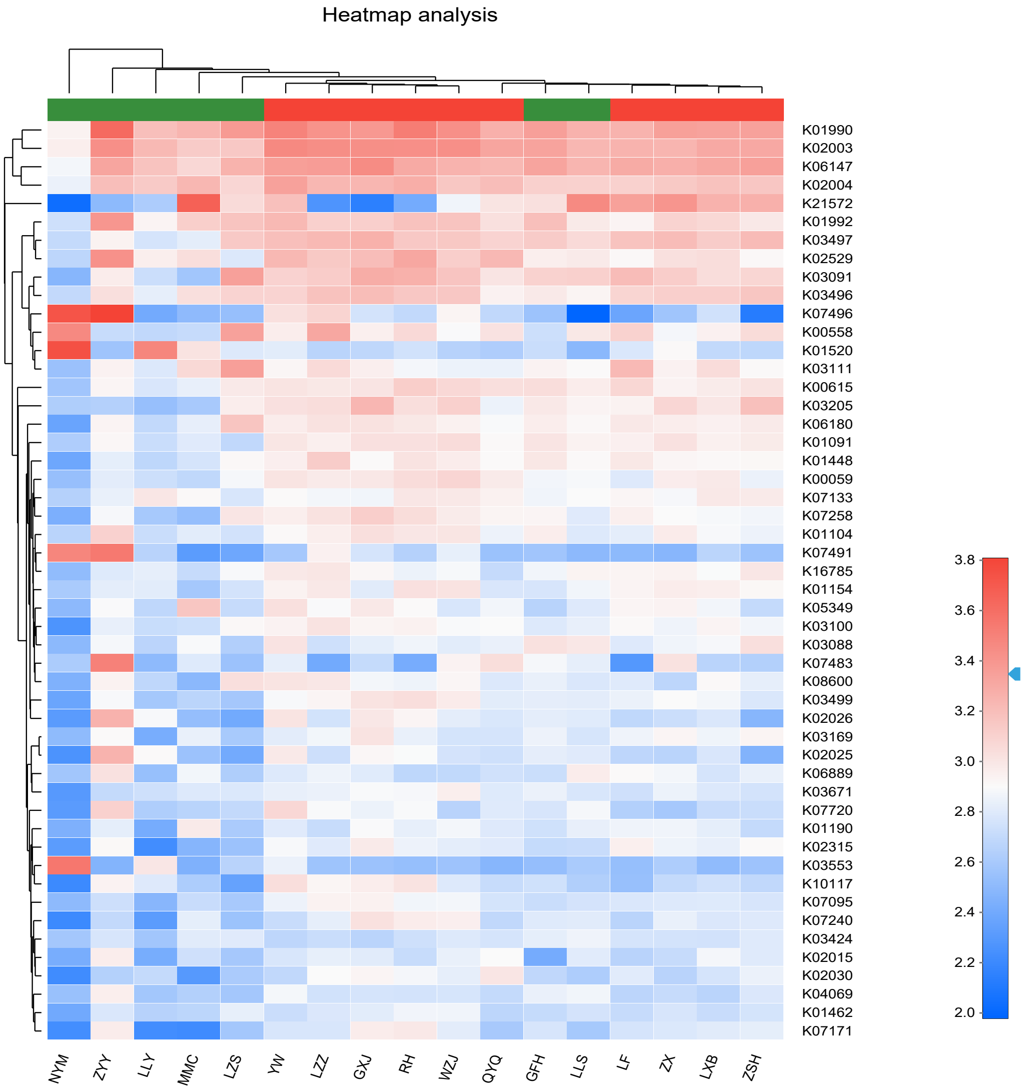

Heatmap analysis
K01990
K02003
K06147
K02004
K21572
K01992
K03497
K02529
K03091
K03496
K07496
K00558
K01520
K03111
K00615
K03205
K06180
K01091
K01448
K00059
K07133
K07258
K01104
K07491
K16785
K01154
K05349
K03100
K03088
K07483
K08600
K03499
K02026
K03169
K02025
K06889
K03671
K07720
K01190
K02315
K03553
K10117
K07095
K07240
K03424
K02015
K02030
K04069
K01462
K07171
NYM
ZYY
LLY
MMC
LZS
YW
LZZ
GXJ
RH
WZJ
QYQ
GFH
LLS
LF
ZX
LXB
ZSH
3.8
3.6
3.4
3.2
3.0
2.8
2.6
2.4
2.2
2.0

Figure S6

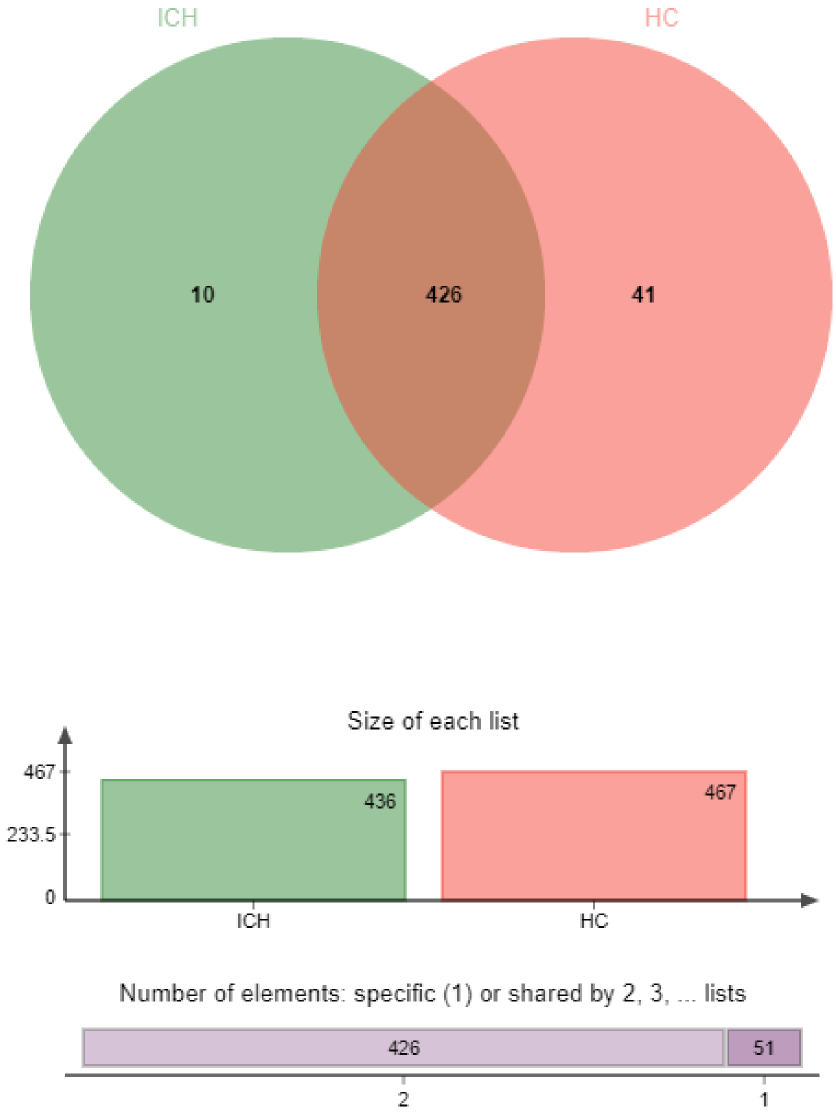
ICH
HC
10
426
41
Size of each list
467
233.5
0
436
467
ICH
HC
Number of elements: specific (1) or shared by 2, 3, ... lists
426
51
2
1

Figure S7

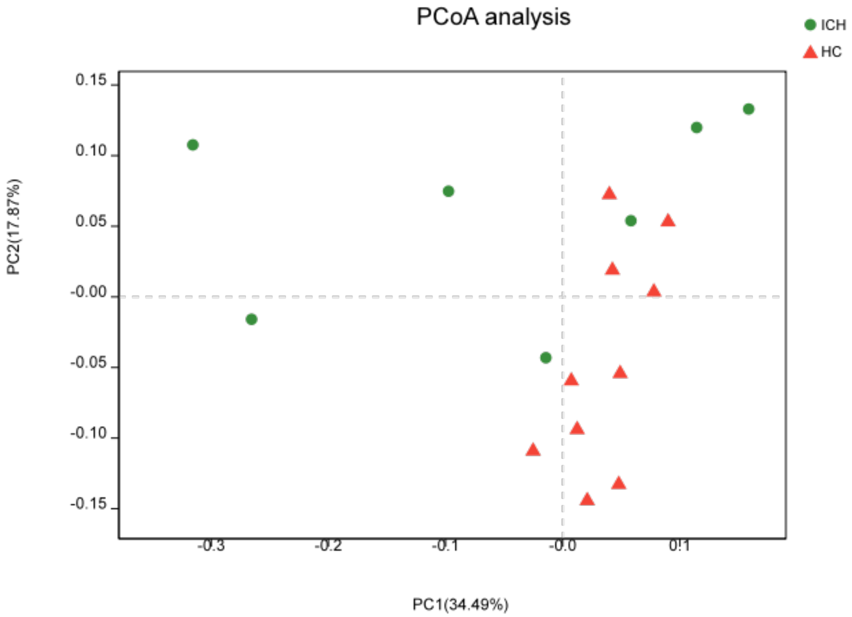
PCoA analysis
ICH
HC
0.15
0.10
0.05
-0.00
-0.05
-0.10
-0.15
-0.3
-0.2
-0.1
-0.0
0.1
PC2(17.87%)
PC1(34.49%)

Figure S8

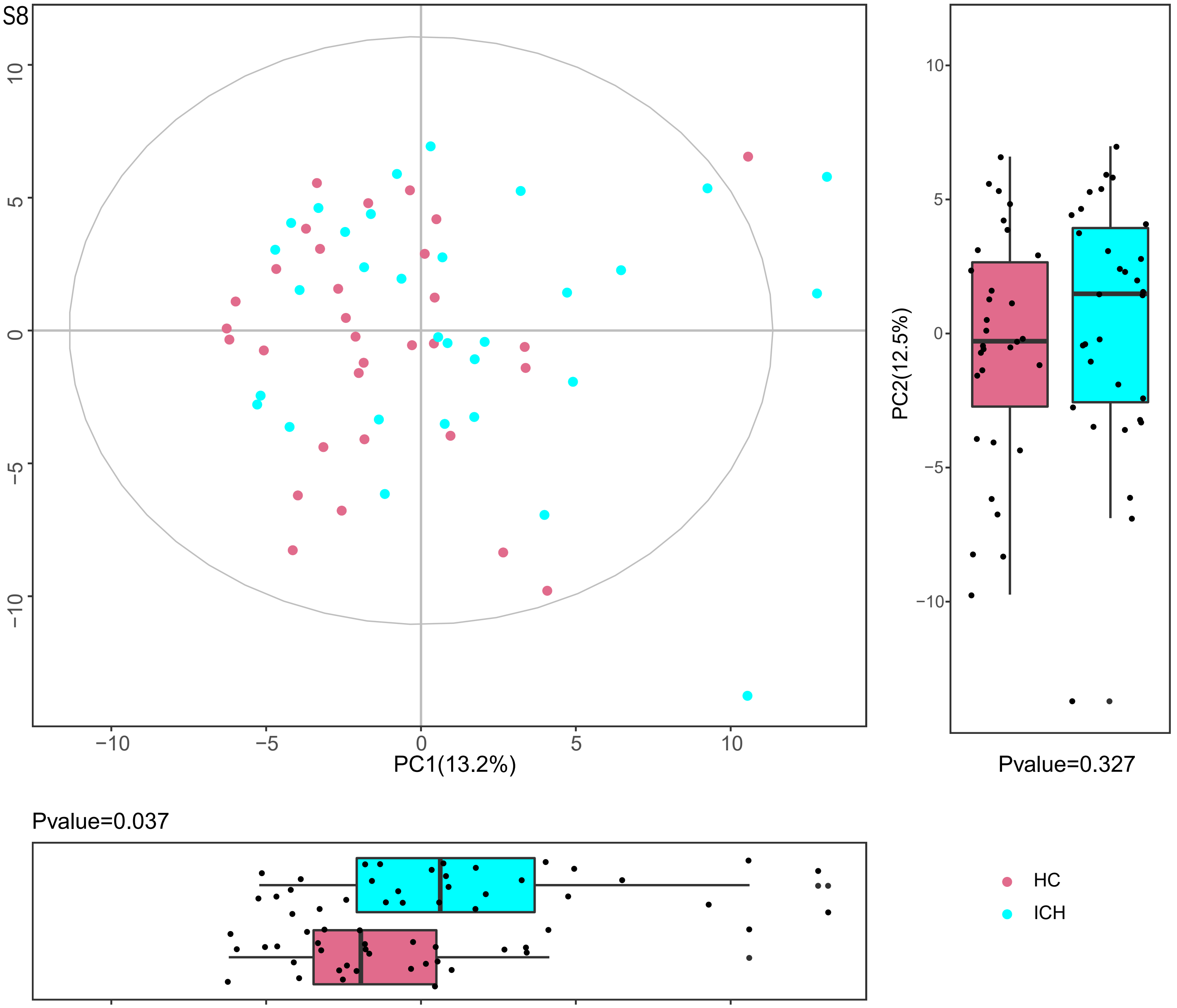

Figure S9

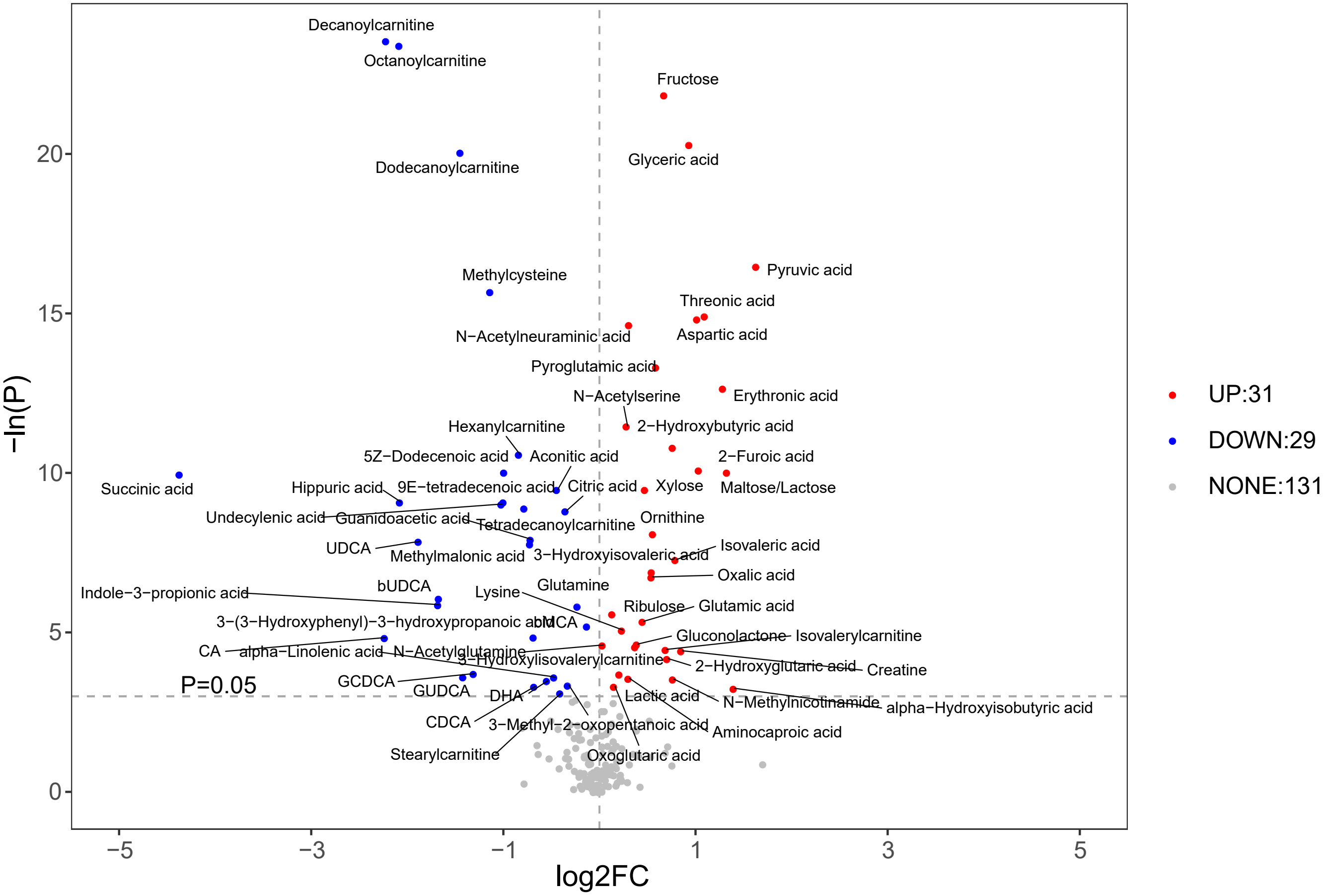

Decanoylcarnitine
Octanoylcarnitine
Fructose
Dodecanoylcarnitine
Glyceric acid
Methylcysteine
Pyruvic acid
Threonic acid
N−Acetylneuraminic acid
Aspartic acid
Pyroglutamic acid
N−Acetylserine
Erythronic acid
Hexanylcarnitine
2−Hydroxybutyric acid
5Z−Dodecenoic acid
Aconitic acid
2−Furoic acid
Succinic acid
Hippuric acid
9E−tetradecenoic acid
Citric acid
Xylose
Maltose/Lactose
Undecylenic acid
Guanidoacetic acid
Tetradecanoylcarnitine
Ornithine
UDCA
Methylmalonic acid
3−Hydroxyisovaleric acid
Isovaleric acid
Oxalic acid
Indole−3−propionic acid
bUDCA
Lysine
Glutamine
Ribulose
Glutamic acid
3−(3−Hydroxyphenyl)−3−hydroxypropanoic acid
bMCA
Gluconolactone
Isovalerylcarnitine
CA
alpha−Linolenic acid
N−Acetylglutamine
3−Hydroxylisovalerylcarnitine
2−Hydroxyglutaric acid
Creatine
P=0.05
GCDCA
GUDCA
DHA
Lactic acid
N−Methylnicotinamide
alpha−Hydroxyisobutyric acid
CDCA
3−Methyl−2−oxopentanoic acid
Aminocaproic acid
Stearylcarnitine
Oxoglutaric acid
−ln(P)
log2FC
0
5
10
15
20
−5
−3
−1
1
3
5
UP:31
DOWN:29
NONE:131

Figure S10

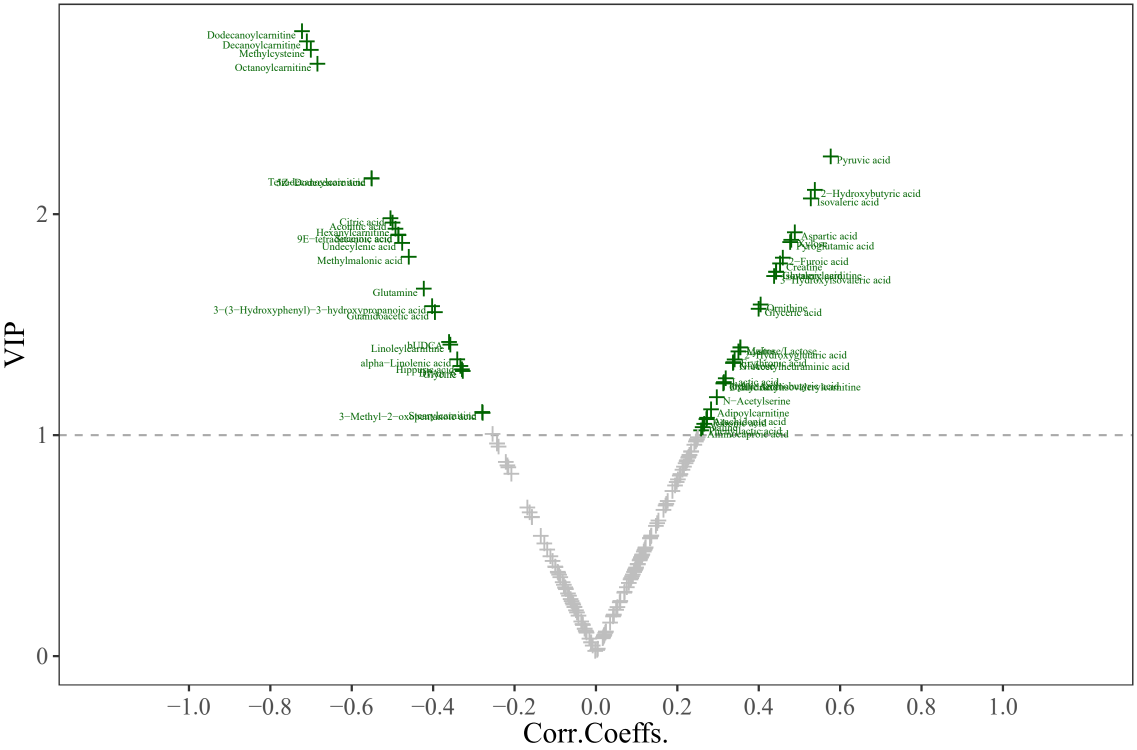

VIP
Corr.Coeffs.
0
1
2
−1.0
−0.8
−0.6
−0.4
−0.2
0.0
0.2
0.4
0.6
0.8
1.0
Dodecanoylcarnitine
Decanoylcarnitine
Methylcysteine
Octanoylcarnitine
Pyruvic acid
2−Hydroxybutyric acid
Isovaleric acid
Citric acid
Hexanoylcarnitine
Undecylenic acid
Methylmalonic acid
Aspartic acid
Pyroglutamic acid
2−Furoic acid
Creatine
3−Hydroxyisovaleric acid
Glutamine
3−(3−Hydroxyphenyl)−3−hydroxypropanoic acid
Guanidoacetic acid
Ornithine
Glyceric acid
bUDCA
Linoleylcarnitine
alpha−Linolenic acid
2−Hydroxyglutaric acid
Erythronic acid
Lactic acid
N−Acetylserine
Adipoylcarnitine
Aminocaproic acid

Figure S1. Microbial composition of ICH and HC groups

(A)Phyla level; (B)Genus level.

Figure S2. Varying distribution of microbiota correlated with the ICH and HC groups

Figure S3. Microbial correlation network

(A)Microbial correlation network among the first 30 species in ICH; (B) Microbial correlation network among the first 30 species in HCs; (C) Microbial correlation network among the first 30 species in all subjects, including ICH and HC.

Figure S4. The shared and unique function KOs of ICH and HC

Figure S5. Heat map of top 50 most abundant KOs

Figure S6. The shared and unique CAZy enzyme genes of ICH and HC

Figure S7. PCoA based on Bray–Curtis difference index shows functional (CAZy enzyme) (β) diversity between samples

Figure S8. PCA based on the relative abundance of serum metabolites showing grouped discrimination

Figure S9. Volcano plot showing the differentially accumulated [log2 (FC) on X-axis] and significantly changed [-ln(p) on Y-axis] serum metabolites of ICH and HC

Figure S10. V plot showing differentially accumulated serum metabolites of ICH and HC from the OPLS-DA models
