# Supplementary material for: Multi-omics reveals specific host metabolism-microbiome associations in intracerebral hemorrhage
Source: Front Cell Infect Microbiol. 2022 Dec 22;12:999627. doi: 10.3389/fcimb.2022.999627 (PMC9813413; doi:10.3389/fcimb.2022.999627)
Supplement: Supplementary file 2 [file DataSheet_2.pdf]

1. Sequence quality controls and DADA2 denoise of 16S rRNA sequencing data

We used QIIME2 to excise the primer fragments of the reads and discard the unmatched primer reads. The DADA2 algorithm was used to perform quality control, denoising, merging and chimera removal. The high-quality reads were summarized to generate a feature table with information about the abundance of ASVs in the samples and removed singletons ASVs. Using the R package, the lengths of all samples containing high-quality reads were counted.

Table S1. Summary statistics for reads volume

Sample	Input	Filtered	Denoised	Merged	Non-chimeric	Non-singleton
LH	103742	93509	89769	80022	46192	44117
NYM	104948	98355	96581	93550	71959	71576
ZCL	114892	108029	105406	100537	60516	59747
YJX	87343	81216	80120	78640	67588	67449
WQH	106041	99497	98034	96240	56175	55814
WQW	102312	95892	93616	89237	60870	60241
WZA	127624	119591	117733	114774	78743	78358
QXG	99541	91713	89518	83397	53234	52484
CLY	109913	102771	101013	95969	70890	70389
WHL	110005	102892	101227	98346	78153	77762
GFH	84649	79150	75719	67865	49811	48973
DXG	167180	149698	146919	141609	99417	98879
WB	124652	116930	115775	114248	105927	105832
GYJ	100847	93811	92132	88312	52997	52392
TJ	105921	99300	97299	93283	67208	66797
XZH	110886	102269	100215	96336	58838	58312
YJW	75557	70331	69366	68590	59193	59095
ZYY	93593	87640	86312	84865	63204	63081
LLS	107486	100596	97221	90056	56871	56073
TJL	96629	89235	86968	82024	52377	51783
XLW	108794	100359	98983	96939	72073	71791
LML	112564	105205	102172	92360	52645	51580
LZS	105964	98847	95094	85780	52651	51654
QXH	99517	91678	89669	85387	54923	54275
HHT	94984	89562	87873	85058	71322	71072
LAH	115106	106954	105405	103151	54998	54609
LLY	111757	103177	101261	98301	77530	77285
LHJ	119052	110709	109621	108470	104134	104061
MMC	114525	106813	105033	101852	71193	70937
FZH	107800	101054	99157	95869	67030	66731
MZS	97468	90033	87806	82860	49029	48430
CLL	108118	100477	98344	94190	66911	66496
WZJ	103052	96422	94032	89129	54640	53939
ZJ	107342	100550	98195	93529	60837	60327
ZDL	105946	98655	96516	91856	74797	74358

LX	101876	95261	93343	88369	54005	53328
LF	109109	101883	98385	88017	52379	51250
RF	102342	95843	93584	88236	61588	60936
RH	104636	96787	94418	89407	55085	54596
LZL	107621	100182	98764	96421	68498	68245
LGR	116217	107605	104587	96499	64064	63313
WMF	116833	109147	106411	99473	68241	67529
YK	114362	107274	105308	101782	66501	66028
GGW	107678	100718	99755	98725	85700	85623
GXJ	111994	105063	102840	97890	67746	67241
ZZ	121229	113460	112278	110393	92352	92222
GXH	100797	93816	91925	87516	61018	60562
LXB	146193	136665	130625	114073	63546	61330
AJJ	126164	118511	116964	114224	95693	95427
JZJ	145410	136871	134977	132472	87324	87045
LBG	128856	120136	118719	116619	81285	81159
QYQ	118185	111354	109027	104594	72125	71544
ZSH	132982	125127	122125	114514	76138	75337
WJ	117985	109539	108240	106844	95425	95273
ZX	125990	116511	114671	110395	65086	64633
ZXN	134500	126018	123964	120504	73536	73228
LH	124976	116647	113468	105823	70400	69383
WL	120846	113088	110097	103477	61080	60260
YW	96900	88826	85911	78800	45193	43253
XJK	139044	130715	129216	127294	87409	87199
LZP	134411	126457	123770	118026	67874	67288
LZZ	137891	128288	127334	126505	124113	124060

Figure 1. Reads length distribution graph

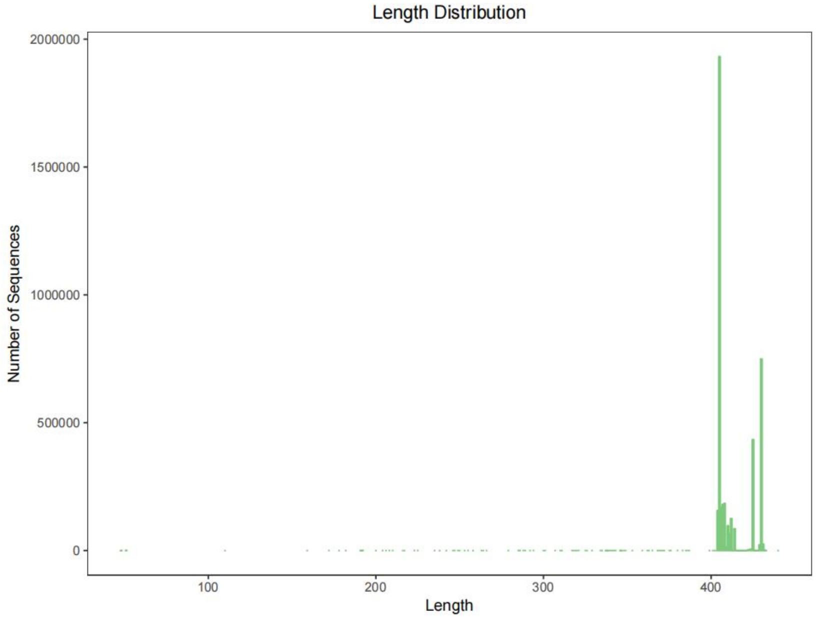

2. Metagenomic sequence analysis

2.1 Sequence quality control

The data were analyzed on the free online platform of Majorbio Cloud Platform (www.majorbio.com). Briefly, the paired-end Illumina reads were trimmed of adaptors, and low-quality reads (length<50 bp or with a quality value <20 or having N bases) were removed by fastp (https://github.com/OpenGene/fastp, version 0.20.0). Reads were aligned to the human genome by BWA (http://bio-bwa.sourceforge.net, version 0.7.9a) and any hit associated with the reads and their mated reads were removed（Table S2）.

2.2 Genome assembly

Metagenomics data were assembled using MEGAHIT (https://github.com/voutcn/megahit, version 1.1.2). Contigs with with a length over 300 bp were selected as the final assembling result, and then the contigs were used for further gene prediction and annotation（Table S3）.

2.3 Gene prediction and non-redundant gene catalog construct

We uesd MetaGene (http://metagene.cb.k.u-tokyo.ac.jp/) to predict open reading frames (ORFs) from each assembled contig. The predicted ORFs with a length over 100 bp were retrieved and translated into amino acid sequences using the NCBI translation table (http://www.ncbi.nlm.nih.gov/Taxonomy/taxonomyhome.html/index.cgi?chapter=tgencodes#SG1). The CD-HIT (http://www.bioinformatics.org/cd-hit/, version 4.6.1) was used to construct a non-redundant gene catalog with 90% sequence identity and 90% coverage. High-quality reads were aligned to the non-redundant gene catalogs to calculate gene abundance with 95% identity using SOAPaligner(http://soap.genomics.org.cn/, version 2.21). We used TPM (Trans Per Million) to evaluate gene abundance in each sample.

Table S2. Removal of host reads statistics

Samples	Optimized reads	Optimized bases (bp)	Percent in raw reads (%)	Percent in raw bases (%)
NYM	65826046	9931115250	73.49568694	73.43196657
LF	70090532	10574785640	74.65320606	74.5905368
LZS	65697476	9913883365	81.07337773	81.02078379
LLS	56810670	8572502094	78.62282393	78.56866608
ZX	60834668	9179585125	78.69435298	78.63909973
LZZ	58577004	8839313734	71.45675722	71.40978895
GXJ	51447380	7760929291	67.83667262	67.77008872
YW	56213212	8480990211	70.92032773	70.86013038
LLY	59117864	8921027277	71.80299985	71.75658715
LXB	61588874	9293765750	74.8636136	74.81407259
ZYY	58085140	8764159748	77.73188683	77.67253993
GFH	62799598	9474213023	71.24311708	71.17905981
RH	54592282	8239221205	74.85455527	74.81629568
MMC	59060742	8912780559	78.49001827	78.44256711
QYQ	63936366	9645190435	78.04999712	77.97561389

WZJ	60032916	9058965887	72.56708981	72.51902303
ZSH	51209112	7726511584	68.54851144	68.49475177

Table S3. Gene prediction result statistic

Sample	ORFs	Total Length(bp)	Average Length(bp)	Max(bp)	Min(bp)
GFH	463804	292975465	631.68	22240	100
YW	289598	204241128	705.26	39876	100
ZYY	142814	96906884	678.55	15246	100
LF	519959	315839528	607.43	18834	100
LZS	461875	268396868	581.1	21672	100
NYM	214872	127273426	592.32	17088	100
RH	286195	182164737	636.51	18720	100
LXB	736728	429070228	582.4	15892	100
QYQ	316541	198382451	626.72	39876	100
WZJ	366454	233003648	635.83	12966	100
ZSH	447339	269963589	603.49	20196	100
LLY	339465	193734986	570.71	17979	100
LLS	303717	197972956	651.83	14730	100
MMC	255205	167513036	656.39	12213	100
LZZ	392676	234614306	597.48	87406	100
ZX	352997	223027208	631.81	15189	100
GXJ	472009	287296639	608.67	21972	100
